# Freshwater wetlands for flood control: How manipulating the hydroperiod affects plant and invertebrate communities

**DOI:** 10.1371/journal.pone.0306578

**Published:** 2024-07-03

**Authors:** Alyssa C. Hockaday, Arturo S. Leon, Kyle Patterson, Steven C. Pennings

**Affiliations:** 1 Department of Biology and Biochemistry, University of Houston, Houston, Texas, United States of America; 2 Department of Civil and Environmental Engineering, Florida International University, Miami, Florida, United States of America; Balochistan University of Information Technology Engineering and Management Sciences, PAKISTAN

## Abstract

Thoughtfully managed hydroperiods in natural and artificial wetlands could potentially provide a combination of desirable flood control services and high ecological functions. To explore how managed freshwater wetlands typical of the Houston, Texas area would respond to different hydrological regimes that might occur if wetlands were drained in anticipation of a heavy rain that did not materialize, we conducted a mesocosm experiment with six flooding depths and seven drought durations, followed by seven months of recovery. We found that the speed in which mesocosms dried out was a function of initial water depth, with mesocosms initially set with greater water depths (30 cm) taking ~ 38 days to dry out versus zero days for wetlands that were completely drained. Individual plant species (14 species planted; 8 species common at the end of the recovery period) were affected by drought length, flooding depth, or their interaction, although details of these responses varied among the species. The composition of the plant community at the end of the drought period was strongly affected by drought length, and the effect of the drought length treatment persisted through seven months of post-drought recovery, with the 80- and 160-day drought treatments diverging most strongly from shorter drought treatments. Above- and below-ground biomass of plants was not affected by the treatments, but above-ground dead biomass (litter) decreased with increasing drought length. Densities of mosquito larvae, snails and tadpoles were temporally variable, and were affected more during the treatment period and early in recovery than after a disturbance event late in recovery. Our results indicate that managed wetlands in southeast Texas would be quite resilient to dry periods of up to 40 days in duration, especially if water was not completely drained at the beginning of the drought. In addition, many species would persist in managed wetlands even with droughts of up to 160 days. This indicates considerable potential for managing the hydroperiods of artificial detention ponds by retaining water longer to increase ecological function, with little to no loss of flood control services, and for managing the hydroperiods of natural wetlands by draining them in advance of anticipated rains to increase flood control services, with little to no loss of ecological function.

## Introduction

Humans strongly alter watershed hydrology by draining natural wetlands, creating impoundments along waterways, and creating off-line detention basins to mitigate effects of heavy rain [1–3]. These alterations affect wetland and riparian biota. Ideally, man-made projects at the watershed scale would have the goal of simultaneously maximizing ecosystem function and flood control benefits for humans.

Detention basins are one common hydrological alteration [4]. Detention basins are ponds designed to temporarily store excess surface runoff and gradually release it over a relatively short period of time to the receiving watercourse [5]. A detention pond has a flow orifice level at the bottom of the pond and does not have a permanent pool of water [6]. Because detention ponds are empty of water most of the year, they provide little to no wetland functions. Alternative designs that held water longer might provide more ecological functions [7, 8], as long as it was possible to drain water in advance of an incoming storm so that flood control function would not be impaired.

In areas that naturally have semi-permanent water bodies, these also could be managed, in this case to increase flood control services by allowing water levels to be dropped in anticipation of a storm that is forecasted to produce flooding. This improved flood control service would come with the risk of reducing ecological functions if the water was drained and a storm did not materialize, such that the wetland dried out.

Central to both these management situations is the need to better understand the risks to ecological function of letting a wetland dry out. This would help determine thresholds for releasing water in advance of incoming storms. Because longer droughts affect ecological communities more than short ones [9], it is likely that drought duration is the key variable determining the risks of “mistakes” in water release.

We take as an example temporary wetlands on the Texas coast. Natural wetlands used to be very abundant throughout the area, but now are mostly drained for agriculture [10]. In urban areas, however, large numbers of detention ponds have been constructed that replace some of the flood control function but little of the ecological function of the lost natural wetlands. Maximizing the function of detention ponds for flood control while at the same time maximizing ecological function would be a desirable goal. Similarly, the remaining natural wetlands could in theory be managed to improve flood control if water levels were dropped in advance of anticipated rains to increase storage capacity. The tradeoff between these objectives hinges on how the ecological function of natural wetlands and detention ponds varies with initial water depth and length of drought. The goal with the research is to identify scenarios that would increase flood control capacity while at the same time increasing functional wetland area—a rare win-win scenario in the struggle to balance human infrastructure with protection of natural systems.

Using a mesocosm study, we explored different scenarios of releasing water from a detention pond or natural water body, draining water to different depths and for different periods of time, to evaluate how aggressively the hydrological regime of artificial and natural wetlands could be manipulated to maximize flood control without severely altering the plant and invertebrate community associated with the wetland. We tested two hypotheses. First, extended droughts would alter the wetland community more than short droughts. In particular, since natural wetlands along the Texas coast typically dry out for part of the year [11], we anticipated that short droughts would have little effect on the wetland community. Second, wetlands that entered into a drought period with a higher initial water level would be more resilient to drought than wetlands that began the drought with little standing water, simply because it would take some time for standing water to be lost to evapotranspiration. In testing these hypotheses, we sought to identify two pieces of information useful to local managers. First, we sought to identify thresholds of drawdown that cause major shifts in the ecological community, allowing us to suggest protocols for drawing down water in wetlands in advance of a storm, and to better understand variation among natural wetlands based on hydroperiod. Second, we sought to rank the various species in the community in terms of sensitivity to drawdown, allowing us to identify which species are most sensitive to hydrological manipulations.

## Materials and methods

We worked at the University of Houston Coastal Center (UHCC), located in central Galveston County, Texas (29.390078, -95.044407). The UHCC is owned and operated by the University of Houston, and research activities were approved by the UHCC Director. The UHCC encompasses about 372.6 ha of gulf coast habitats utilized for research, education, and services related to the environment since 1961 [12]. Soil, plants and invertebrates used in the experiment were collected from man-made wetlands on the property, and so were appropriate for Galveston County. To compare our experimental treatments with natural hydroperiods for the area, two pressure sensors (HOBO Water Level (30 ft) Data Logger U20L-01) were deployed in shallow (1–3 m deep) man-made ponds on the property between December 2, 2019 and October 25, 2021.

To conduct the experiment, we deployed 40 round mesocosms (1.5 m in diameter, 1 m deep), arranged in four rows of ten, under a clear plastic roof to exclude rainfall (S1 Fig). Soil (Lake Charles clay, 0.13 percent nitrogen by dry mass) was collected from a drainage ditch spoil pile on the property. We used this soil to fill each mesocosm to a depth of approximately 30 cm, and flooded the soil with water. From January 9, 2019 to February 19, 2019, we added fourteen species of local plants, collected from wetlands on the property, to the mesocosms. This also added propagules of these and other species that were carried within the soil blocks, such that a fifteenth species colonized on its own (Table 1). All plant species were native except for *Ludwigia hexapetala*. All plant species, other than the two *Eleocharis* species, will be referred to by genus hereafter. Water pumped from a nearby well was added to the mesocosms to flood them to 15 cm above the soil surface. At this point, vegetation was allowed to establish and grow for an entire year (the “growth period”) with the water level maintained at 15 cm above the soil surface. In February 2020 we relocated some plants among individual mesocosms to better standardize the abundance of each plant species in each mesocosm. In April 2020, six small snails (not identified to species) collected from wetlands on the property were added to each of the tanks. For the duration of the experiment, the tops of the mesocosms were open to the air, allowing colonization by plant propagules, invertebrates and amphibians. In May 2020, at the end of the one year growth period, the plants had spread to thickly fill the mesocosms, and snails and aquatic insects were present in the standing water.

**Table 1:** Plant species measured in the mesocosms.

Family and Species	Common Name	Wetland Indicator	Abundance Metric
*Sagittaria platyphylla* (Alismataceae)	delta arrowhead	OBL	individual culm
*Hydrocotyle verticillata* (Araliaceae)	whorled marshpennywort	OBL	percent cover
*Solidago sempervirens* (Asteraceae)	seaside goldenrod	FACW	individual culm
*Symphyotrichum subulatum* (Asteraceae)	eastern annual saltmarsh aster	OBL	individual culm
*Ceratophyllum demersum* (Ceratophyllaceae)	coon’s tail	OBL	percent cover
*Cyperus ochraceus* (Cyperaceae)	pond flatsedge	FACW	individual culm
*Eleocharis cellulosa* (Cyperaceae)	Gulf Coast spikerush	OBL	percent cover
*Eleocharis montevidensis* (Cyperaceae)	sand spikerush	FACW	percent cover
*Schoenoplectus tabernaemontani* (Cyperaceae)	softstem bulrush	OBL	individual culm
*Juncus brachycarpus* (Juncaceae)	whiteroot rush	FACW	individual culm
*Ludwigia hexapetala* (Onagraceae)	creeping waterprimrose	OBL	percent cover
*Bacopa monnieri* (Plantaginaceae)	herb of grace	OBL	percent cover
*Panicum hemitomon* (Poaceae)	maidencane	OBL	individual culm
*Typha domingensis* (Typhaceae)	southern cattail	OBL	individual culm
*Phyla nodiflora* (Verbenaceae)	turkey tangle fogfruit	FAC	percent cover

*Ceratophyllum* colonized the mesocosm on its own; the other 14 species were planted. All species were native except for *Ludwigia*. Common names and wetland indicator status are from the 2016 USDA National Wetland Plant List for the Atlantic and Gulf Coastal Plain region [13]. The wetland indicator status for *Ludwigia* was not listed and so was assigned based on other species in the genus, all of which were OBL. Wetland Indicator Statuses are as follows: OBL = obligate wetland, FACW = facultative wetland, FAC = facultative, FACU = facultative upland and UPL = upland. The “Abundance Metric” denotes how each plant species was measured throughout the experiment. Counts of individual culms were used for species that occurred as discrete individuals or species with multiple shoots or leaves extending up from belowground rhizomes. Estimates of percent cover were used for low-stature species that spread across the soil surface.

**Fig 1:**
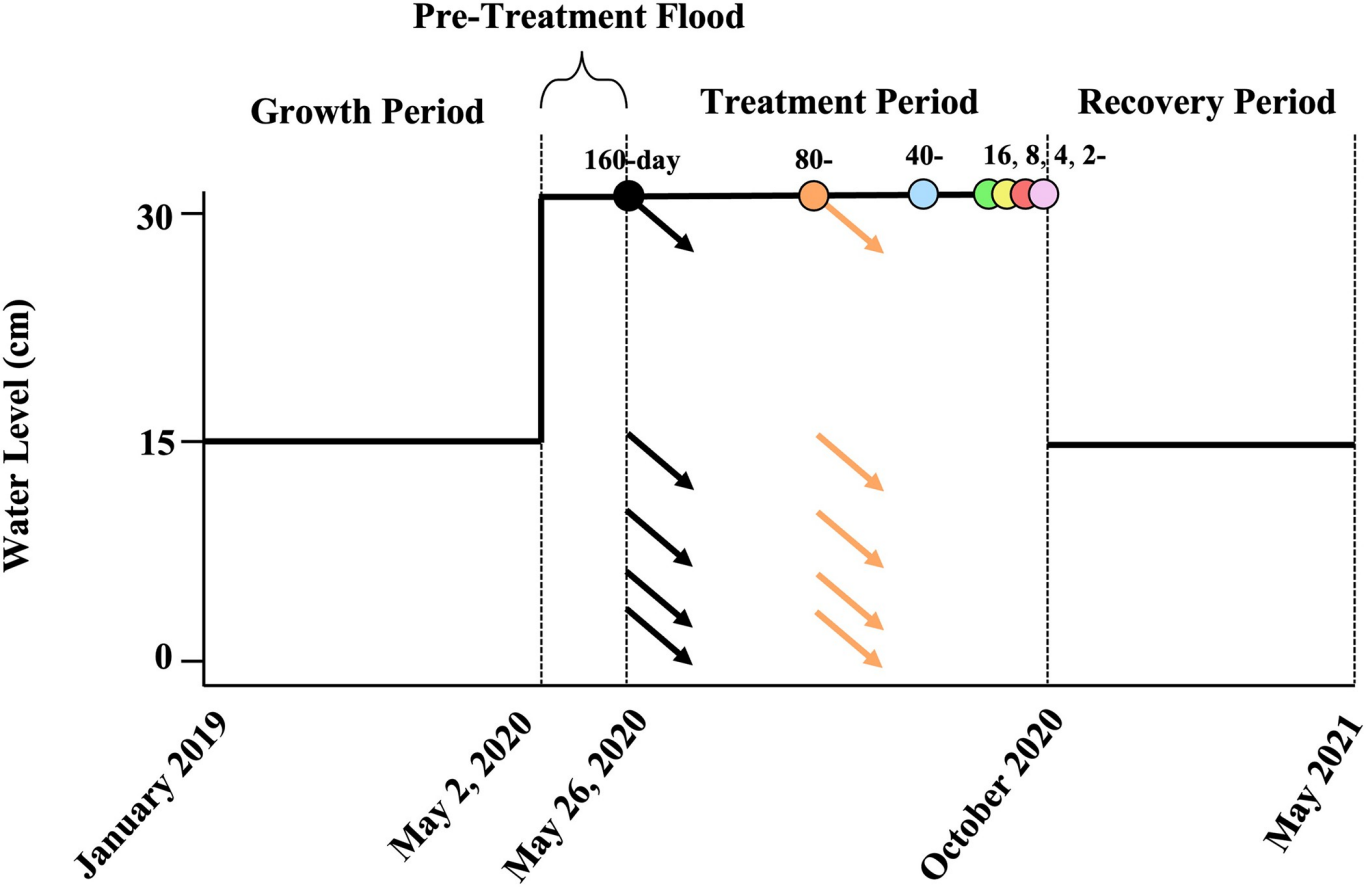
Timeline of the experiment. During the 16 month-long growth period, all mesocosms were flooded to 15 cm water depth while plants spread from initial plantings throughout the mesocosms. During the 24-day pre-treatment period, all mesocosms were flooded to 30 cm water depth to initiate the start of the treatments. In late May 2020, the first drawdown period began and lasted for 160-days, then 80 days later the 80-day duration drawdown period began, followed in turn by the 40-, 16-, 8-, 4- and 2-day drawdown periods. Each drawdown duration had multiple water level depths. For the 160- and 80-day drawdowns, arrows indicate the starting water depths and then the general trajectories of declining water depth thereafter due to evapotranspiration. For clarity, arrows are not shown for the shorter drawdown periods. All treatment durations ended on the same day in October 2020 when the recovery period began. During the 7-month recovery period, all mesocosms were maintained at 15 cm water depth until the harvest in May 2021.

We began a 24-day pre-treatment period on May 2, 2020. On this day, all mesocosms were flooded to 30 cm water depth to mimic wet conditions. Near the end of the month, on May 26, 2020, the treatment period began in which water drawdowns were conducted to mimic a full range of possible wetland conditions, from permanent (always wet) to very dry (similar to shallow depressional wetlands or detention ponds). In order for each of the treatments to end on the same day, the longest drawdown treatments began at the start of the treatment period, whereas shorter drawdown treatments began later in the summer. For example, the first drawdown treatments began on May 26, 2020 with the 160-day duration, then 80 days later for the 80-day duration, and so forth (Fig 1). Each mesocosm was an independent treatment combination, with water depth initially set to 0, 2, 4, 7, 15, or 30 cm (i.e., 30 cm = no drawdown; water maintained) and water depth allowed to naturally decline further after the initial drawdown due to evapotranspiration for 2, 4, 8, 16, 40, 80, or 160 days. Because we had only 40 mesocosms, two treatment combinations (15 cm/8 days; and 15 cm/160 days) were omitted to give a total of 40 replicates (6 drawdown depths x 7 durations = 42, minus 2 omitted treatment combinations = 40), with each treatment combination replicated once. During the treatment period, the soil in the mesocosms subjected to the longer drawdown durations became dry and cracked at the surface. At the end of the treatment period, on October 21, 2020, the recovery period began. We returned water levels in all 40 mesocosms to 15 cm and maintained it at that level for the following seven months to allow the mesocosms to recover (Fig 1).

We measured soil conditions, water conditions, plant abundance and animal abundance throughout the experiment. We measured water depth weekly during the treatment period with a ruler, water temperature with an electronic thermometer, soil temperature (top 10 cm) with an electronic thermometer, and soil water content gravimetrically. These measurements continued at ~monthly intervals throughout the recovery period. In addition, a single temperature logger (HOBO Water Temperature Pro v2, U22-001) was deployed (oriented vertically, with mounting hole ~10 cm below the soil surface and the optical interface ~25 cm below the soil surface) in the center of each mesocosm on May 11, 2020, before the treatment period began, to measure soil temperature at 30-minute intervals over the duration of the experiment. An additional temperature logger (same model) was deployed (oriented vertically) hanging from a support beam within the greenhouse to measure ambient air temperatures at 30-minute intervals over the duration of the experiment.

We measured plant composition (percent cover or individual culms depending on species) monthly for each species present in the mesocosms (Table 1). We estimated density of three common animals monthly. Snails and frogs (categorized into four life-history stages: tadpole, tadpole with two legs, tadpole with four legs and adult frog) were counted in the entire mesocosm. Only frogs in the first life-history stage were present in notable quantities. Mosquito larvae were counted within a 10 cm x 10 cm quadrat placed haphazardly within the mesocosm.

At the end of the 7-month recovery period on May 18, 2021, we conducted a final round of sampling of all variables as described above. Finally, from May 19–21, 2021, we harvested the plants from the mesocosms. Aboveground plant biomass was clipped from a 0.5 x 0.5 m quadrat placed in the center of each mesocosm (S2 Fig), dried at 60 degrees Celsius for 5 days, sorted to species, and weighed. Belowground biomass was measured by collecting a single monolith (~ 17.5 cm deep, 10 cm wide, 10 cm long) from each tank within the area harvested for aboveground biomass. Soil was washed away over a sieve and the remaining roots and rhizomes were dried and weighed (all species combined). All data are available online [14].

Data were analyzed with multiple regression, with drought length, water depth, and their interaction as the independent variables. We conducted separate analyses for the end of the treatment period, the end of the recovery period, and (for plants) the harvest data at the end of the recovery period. Because animal abundance varied considerably over time during the treatment period, we selected a date during the treatment period for each animal species when it was most abundant across all treatments, and analyzed data at this date rather than at the end of the treatment period. In addition, we compared plant community composition across the drought length treatments (because drought length was generally more important than water depth in the analyses of individual species) using NMDS. Again, we conducted separate analyses for the end of the treatment period, the end of the recovery period and the harvest data at the end of the recovery period. The nonmetric multidimensional scaling (NMDS) ordination is a representation of dissimilarities among treatments based on the Bray-Curtis similarity matrix, square root transformed. All statistical analyses were completed in R 4.0.3.

## Results

### Abiotic factors

**Fig 2:**
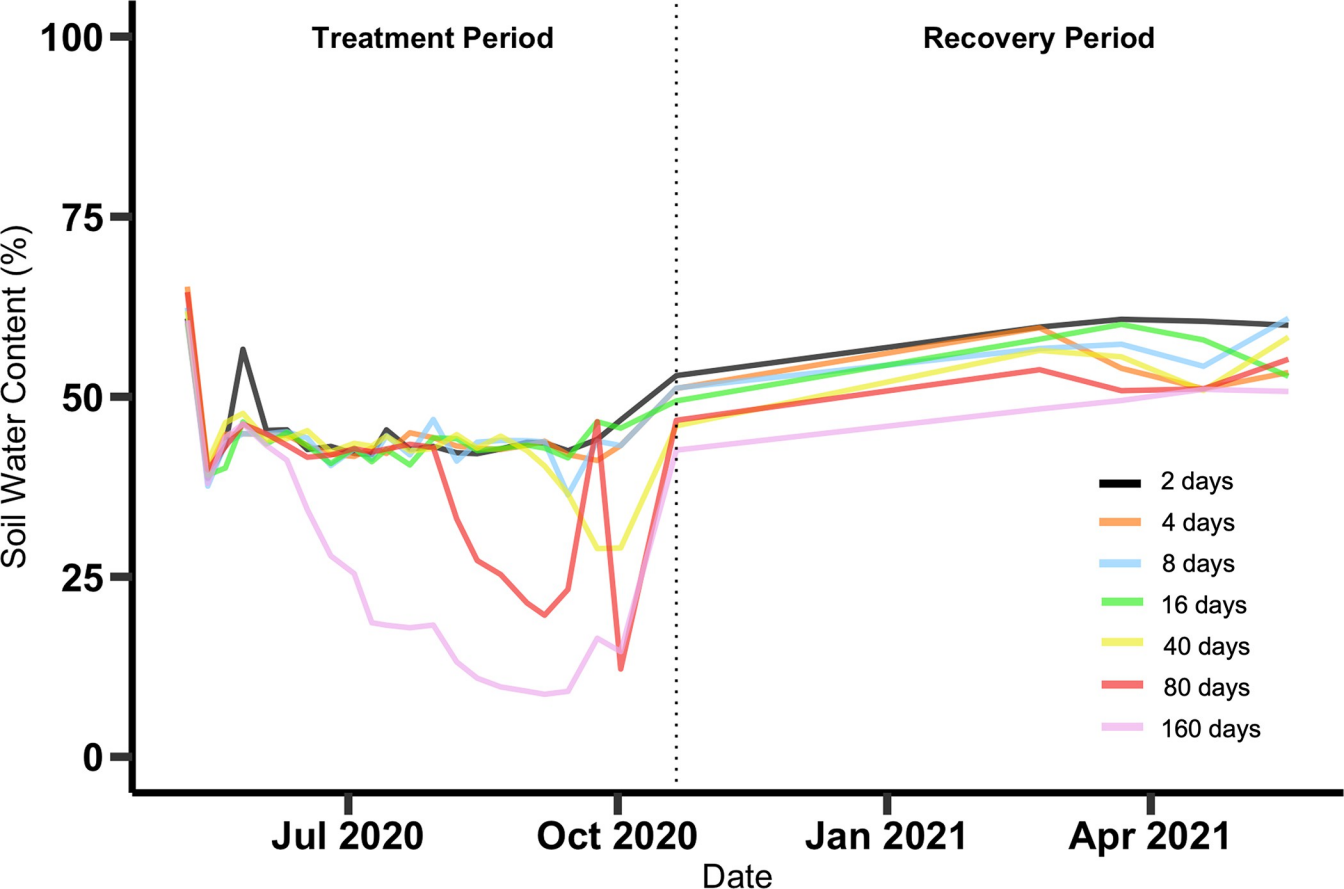
Soil water content. Soil water content (percent of the mass of a wet soil sample consisting of water) overtime, averaged across water depths for each drought period.

Air temperatures were warm (~25–40 degrees C) during the treatment period in the summer of 2020 and cool (~0–25 degrees C) during the recovery period in the winter of 2020–21 (S3A Fig). Air temperatures dropped below freezing on eight days during the recovery period, with three brief dips just below freezing in December 2020 and January 2021, followed by five days with harder and more extended freezes between February 14 and 19, 2021. Soil and water temperatures followed the same seasonal pattern but were less variable, never getting as warm as air temperatures and never dropping below freezing (S3B–S3D Fig). Soil water content, expressed as the percent of soil wet weight that consisted of water, dipped during the drought periods and increased slightly by about 10 percent over the course of the recovery period (Fig 2). Water depth approximated the nominal treatment depths during the pre-treatment period, at the start of the treatment period, and during the post-treatment period (S4 Fig). During the treatment periods, water depth gradually dropped in the mesocosms due to evapotranspiration. Water depth did not drop to zero (soil level) in most of the shorter drought length treatments, and the mesocosms that started with greater water depths took longer to dry out than those starting with smaller water depths. The mesocosms with the greatest initial water depth, 30 cm, took 32–46 days to dry out in the different drought duration treatments. The hydrological regimes that we imposed on the mesocosms were reasonable given natural hydroperiods in the area: water levels recorded by pressure sensors in the two ponds near the experimental location dropped by ~15 cm over ~15-day periods between major rain events that rapidly raised water level by ~20 cm (S5 Fig).

### Plants

Four of the fourteen plants species that we added at the start of the experiment (*Sagittaria*, *Symphyotrichum*, *Ludwigia* and *Panicum*) did not survive to the treatment period. Another three (*Solidago*, *Cyperus* and *Phyla*) survived but were rare across all treatments regardless of drought length or water depth. These seven species were not analyzed separately, but if present were included in the NMDS analysis of community composition.

**Fig 3:**
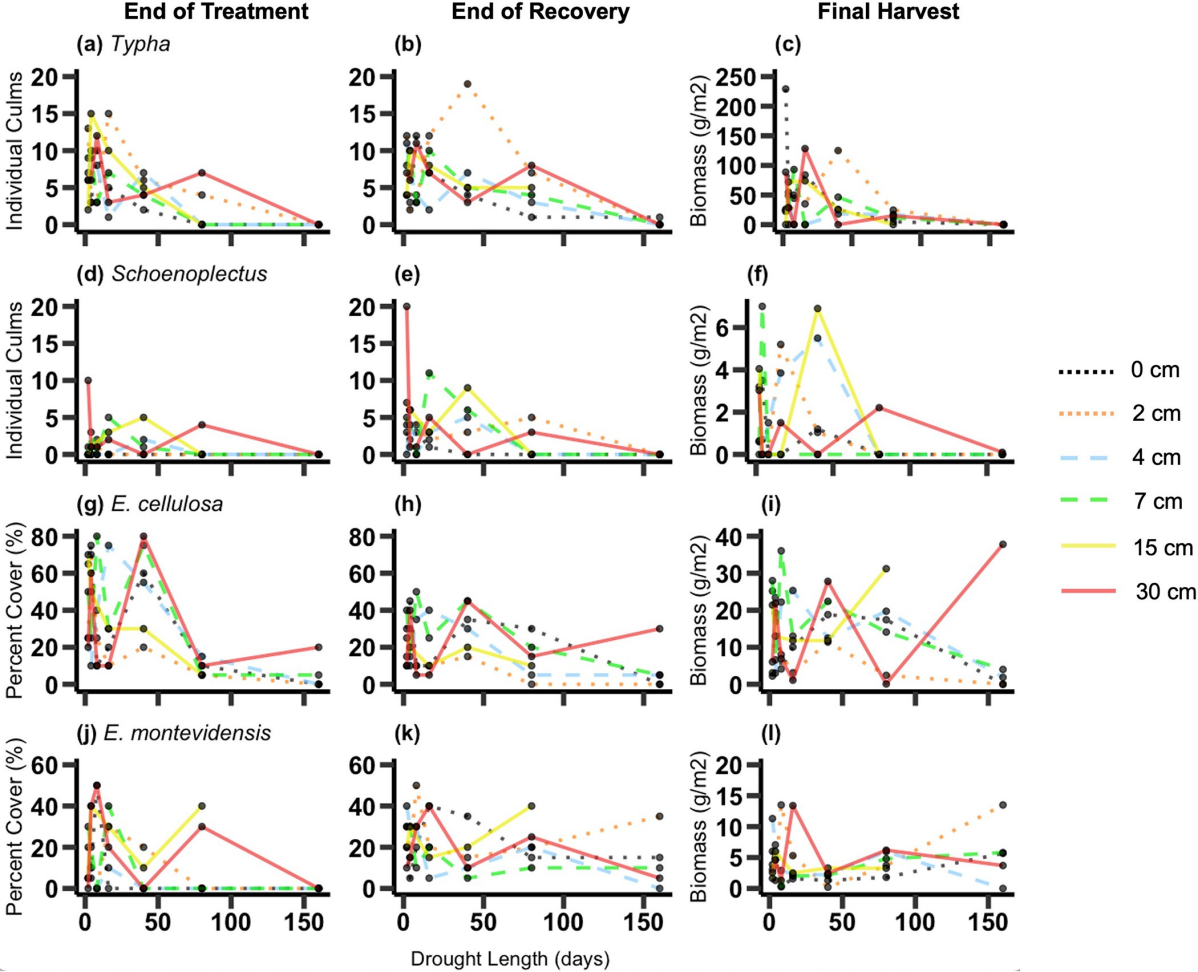
Plant abundance Part 1. Plant abundance for *Typha* (a-c), *Schoenoplectus* (d-f), *E*. *cellulosa* (g-i) and *E*. *montevidensis* (j-l) as a function of drought length and water depth. The first column of panels shows plant abundance at the end of the treatment period, the second column shows the end of the recovery period and the last column shows the biomass (g/m^2^) at the final harvest. Statistical results are shown in Table 2.

**Fig 4:**
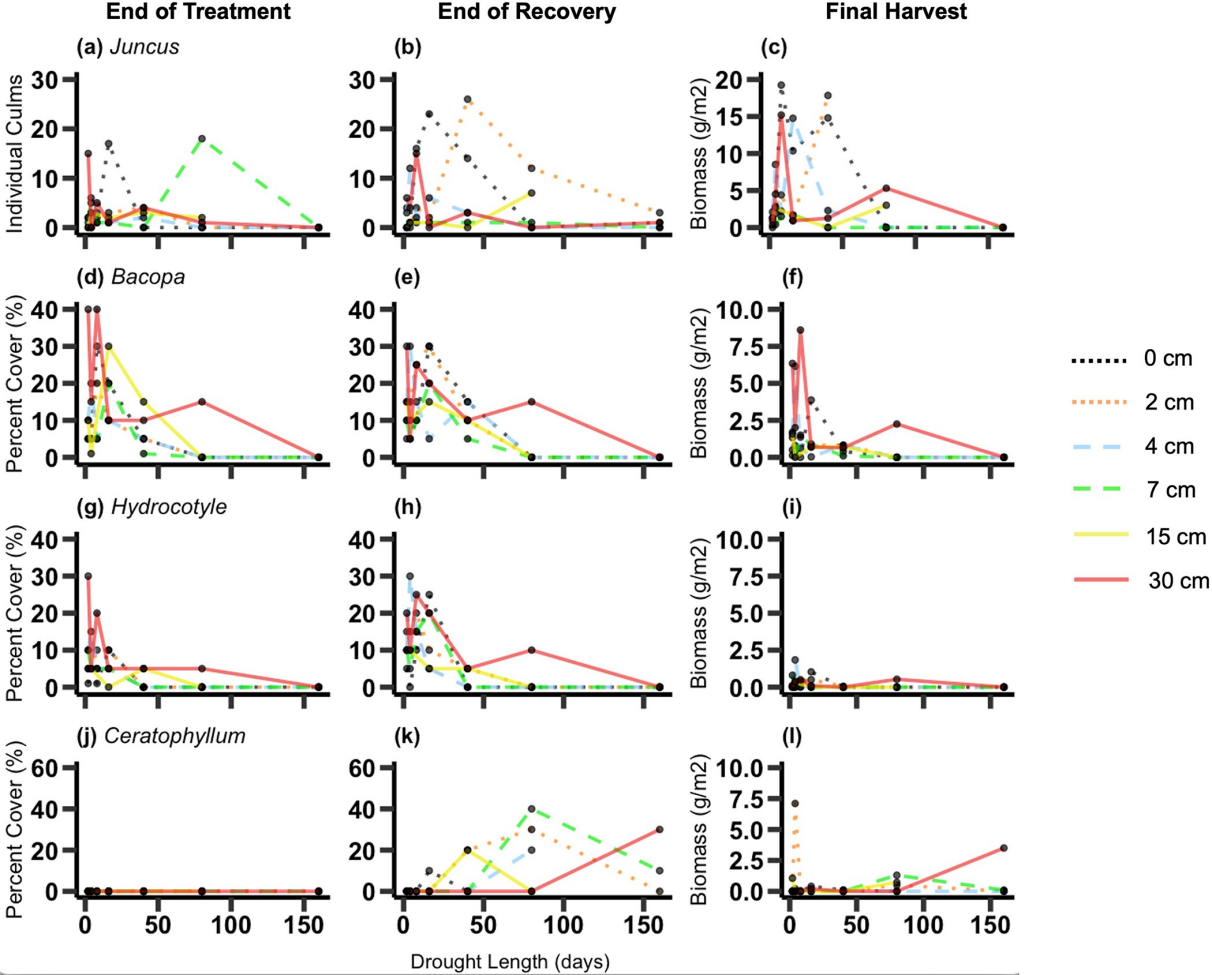
Plant abundance Part 2. Plant abundance for *Juncus* (a-c), *Bacopa* (d-f), *Hydrocotyle* (g-i) and *Ceratophyllum* (j-l) as a function of drought length and water depth. The first column of panels shows plant abundance at the end of the treatment period, the second column shows the end of the recovery period and the last column shows the biomass (g/m^2^) at the final harvest. Statistical results are shown in Table 2.

Across all plant species, abundance at the end of the treatment period was affected by both drought length and water depth, but abundance ay the end of the recovery period was mostly affected by drought length (Table 2). *Typha* culm count was reduced by longer drought lengths both at the end of the treatment period (*p* = 0.0005) and at the end of the recovery period (*p* = 0.006). Biomass at the final harvest followed the same trend but was not statistically significant (Fig 3A–3C and Table 2). *Schoenoplectus* culm count at the end of the treatment period was only affected by water depth (*p* = 0.005), with lowest counts in the treatments starting with shallow water depths. Its culm count and biomass did not vary among treatments at the final harvest (Fig 3D–3F and and Table 2). The cover of *E*. *cellulosa* was decreased by longer drought lengths at the end of the treatment period (*p* = 0.002) and also at the end of the recovery period (*p* = 0.004). Its biomass was generally reduced by longer drought lengths, but increased with drought length for the deeper water treatments, leading to a significant drought length x water depth interaction (drought length: *p* = 0.02; drought length x water depth: *p* = 0.008) (Fig 3G–3I and Table 2). The cover and biomass of *E*. *montevidensis* was not affected by the treatments (Fig 3J–3L; Table 2). Similarly, the culm count and biomass of *Juncus* was not affected by the treatments (Fig 4A–4C and Table 2). *Bacopa* was affected by different factors in each analysis. At the end of the treatment period, its cover was lowest at longer drought lengths (*p* = 0.05) and shallower water depths (*p* = 0.005). At the end of the recovery period, *Bacopa* cover was still reduced by longer drought lengths (*p* = 0.002), but no longer by the history of water depth treatments. Its biomass, however was not affected by drought length but was reduced by past shallower water depths (*p* = 0.006) (Fig 4D–4F and Table 2). The cover of *Hydrocotyle* at the end of the treatment period was reduced in the treatments with shallower water depths (*p* = 0.006), but at the end of the recovery period in treatments with longer drought lengths (*p* = 0.003). There was, however, no significant effect of treatments on the biomass of *Hydrocotyle* at the final harvest (Fig 4G–4I and Table 2). *Ceratophyllum* did not invade the mesocosms until the recovery period. At the end of the recovery period, its cover (*p*<0.0001) and biomass (*p* = 0.004) generally increased with longer drought lengths (Fig 4J–4L and Table 2).

**Table 2:** Plant abundance.

**End of Treatment**												
**Species**	**Variables**	**Std. Error**		**t-value**		**R^2^**		***df***	**F**			***p*-value**
*Typha*	Drought Length	0.01		-3.78		0.39		36	7.62			**0.0005**
	Water Depth	0.07		0.26								0.8
	DL x WD	0.001		0.21								0.84
*Schoenoplectus*	Drought Length	0.007		-0.42		0.25		36	4.02			0.68
	Water Depth	0.04		2.96								**0.005**
	DL x WD	0.0005		-1.11								0.28
*E*. *cellulosa*	Drought Length	0.09		-3.36		0.28		36	4.61			**0.002**
	Water Depth	0.46		-0.58								0.56
	DL x WD	0.007		0.92								0.36
*E*. *montevidensis*	Drought Length	0.06		-1.63		0.21		36	3.25			0.11
	Water Depth	0.29		1.72								0.09
	DL x WD	0.004		-0.28								0.78
*Juncus*	Drought Length	0.18		-0.52		0.05		36	0.68			0.61
	Water Depth	0.09		0.86								0.40
	DL x WD	0.001		-0.55								0.59
*Bacopa*	Drought Length	0.03		-2.03		0.40		36	7.89			**0.05**
	Water Depth	0.17		3.01								**0.005**
	DL x WD	0.002		-1.25								0.22
*Hydrocotyle*	Drought Length	0.02		-1.95		0.39		36	7.71			0.06
	Water Depth	0.10		2.93								**0.006**
	DL x WD	0.001		-1.42								0.16
**End of Recovery**												
**Species**	**Variables**		**Std. Error**		**t-value**	**R** ^**2**^	***df***		**F**		***p-value***	
*Typha*	Drought Length		0.01		-2.93	0.29	36		4.84		**0.006**	
	Water Depth		0.07		0.12						0.91	
	DL x WD		0.001		0.006						0.99	
*Schoenoplectus*	Drought Length		0.01		-1.43	0.23	36		3.60		0.16	
	Water Depth		0.07		1.79						0.08	
	DL x WD		0.001		-1.05						0.30	
*E*. *cellulosa*	Drought Length		0.05		-3.06	0.21	36		3.15		**0.004**	
	Water Depth		0.26		-1.03						0.31	
	DL x WD		0.004		1.93						0.06	
*E*. *montevidensis*	Drought Length		0.05		-1.22	0.09	36		1.14		0.23	
	Water Depth		0.23		-0.16						0.87	
	DL x WD		0.003		-0.25						0.80	
*Juncus*	Drought Length		0.03		-0.75	0.05	36		0.65		0.46	
	Water Depth		0.13		-0.68						0.50	
	DL x WD		0.002		-0.08						0.94	
*Bacopa*	Drought Length		0.03		-3.44	0.39	36		7.55		**0.002**	
	Water Depth		0.15		0.96						0.35	
	DL x WD		0.002		-0.17						0.86	
*Hydrocotyle*	Drought Length		0.03		-3.25	0.39	36		7.82		**0.003**	
	Water Depth		0.13		1.41						0.17	
	DL x WD		0.002		-0.41						0.68	
*Ceratophyllum*	Drought Length		0.06		5.16	0.49	36		11.67		**<0.0001**	
	Water Depth		0.31		-0.30						0.77	
	DL x WD		0.004		-1.49						0.14	
**Biomass at Final Harvest**												
**Species**	**Variables**		**Std. Error**		**t-value**	**R** ^**2**^		***df***		**F**		***p-value***
*Typha*	Drought Length		0.18		-1.98	0.15		36		2.19		0.06
	Water Depth		0.88		-0.27							0.79
	DL x WD		0.01		0.03							0.98
*Schoenoplectus*	Drought Length		0.01		-1.62	0.08		36		1.08		0.11
	Water Depth		0.04		-0.32							0.75
	DL x WD		0.0006		0.43							0.67
*E*. *cellulosa*	Drought Length		0.04		-2.51	0.21		36		3.14		**0.02**
	Water Depth		0.19		-1.14							0.26
	DL x WD		0.003		2.80							**0.008**
*E*. *montevidensis*	Drought Length		0.01		0.80	0.02		36		0.24		0.43
	Water Depth		0.07		0.52							0.60
	DL x WD		0.001		-0.49							0.63
*Juncus*	Drought Length		0.03		-0.66	0.04		36		0.54		0.51
	Water Depth		0.14		-0.72							0.48
	DL x WD		0.002		-0.01							0.99
*Bacopa*	Drought Length		0.007		-0.99	0.30		36		5.02		0.33
	Water Depth		0.03		2.92							**0.006**
	DL x WD		0.0005		-1.39							0.17
*Hydrocotyle*	Drought Length		0.001		-1.67	0.09		36		1.12		0.10
	Water Depth		0.007		-0.30							0.77
	DL x WD		0.00005		0.50							0.62
*Ceratophyllum*	Drought Length		0.06		3.09	0.23		36		3.51		**0.004**
	Water Depth		0.28		0.45							0.66
	DL x WD		0.004		-1.82							0.08

Results from multiple linear regression analyses of drought length and water depth and their interaction on plant abundance at the end of treatment, end of recovery and the final harvest. No analysis is shown for *Ceratophyllum* at the end of the treatment period because it did not appear until the recovery period. Significant *p*-values <0.05 are shown in bold font. DL: drought length; WD: water depth.

**Fig 5:**
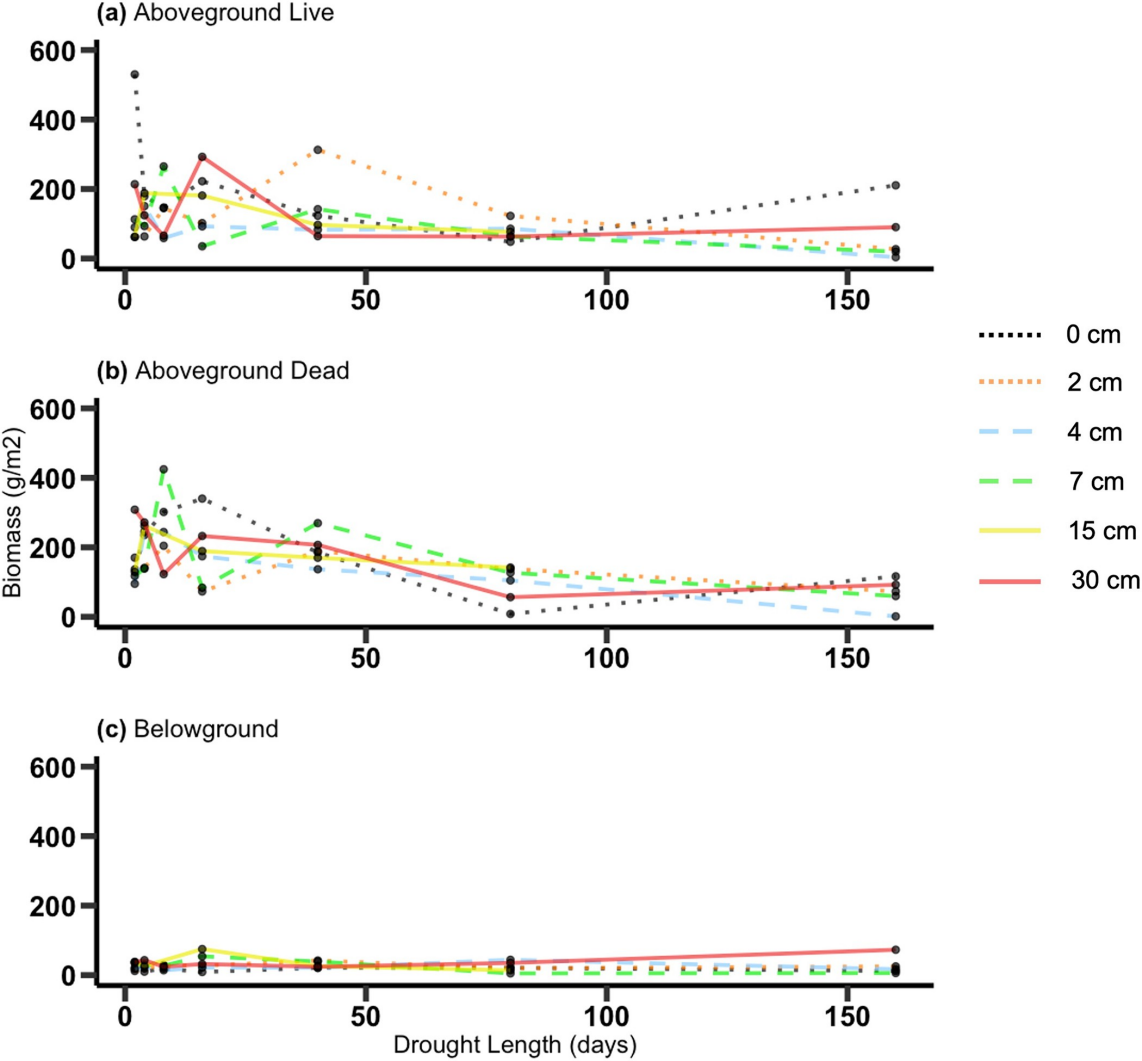
Final harvest. Aboveground total live biomass (a), aboveground dead biomass (b) and belowground total live biomass (c) (g/m^2^) as a function of drought length and water depth.

At the final harvest there was no effect of treatments on the total live aboveground biomass or total belowground biomass of the plants (all species grouped). Dead aboveground biomass, however, decreased with longer drought lengths (*p* = 0.007) (Fig 5 and S1 Table).

**Fig 6:**
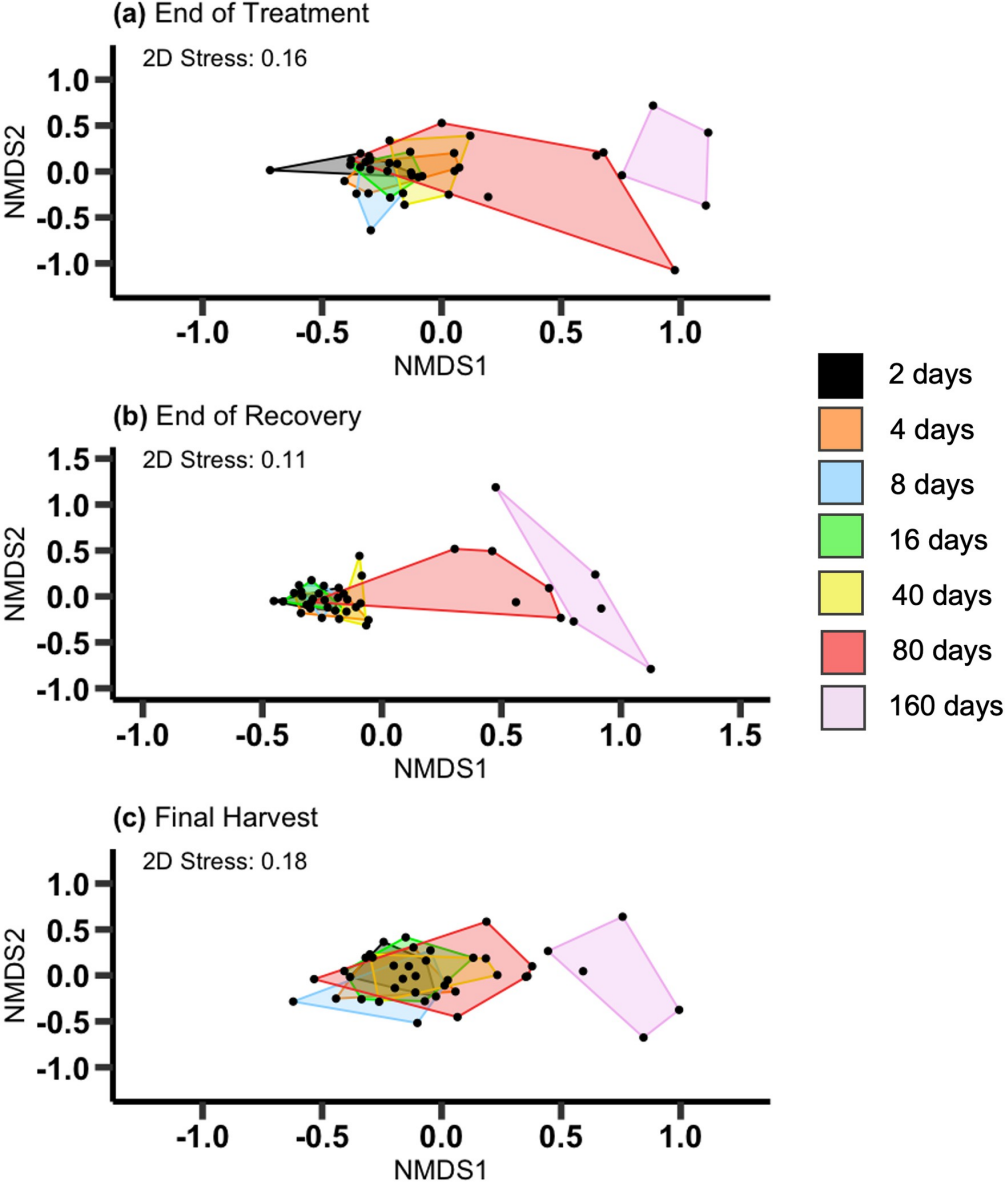
NMDS of plant community. NMDS ordination of plant community composition as a function of drought length. Separate analyses were conducted for the end of treatment, end of recovery and final harvest. Abundance metrics used in the NMDS for panel a and b followed the abundance metrics used for each species (Table 1) and the abundance metric used in panel c was plant biomass (g/m^2^). 2D stress values were less the 0.20 indicating results do not have to be interpreted with caution.

The NMDS analyses indicated that the composition of the plant communities differed across drought lengths (Fig 6). At the end of the treatment and recovery periods, the 80-day and 160-day treatments had relatively little overlap with the shorter drought periods, all of which overlapped with each other (Fig 6A, 6B and S2 Table; End of Treatment: PERMONOVA, *p* = 0.001; End of Recovery: PERMONOVA, *p* = 0.001). The analysis based on aboveground biomass at the final harvest showed a similar trend, but only the 160-day treatment was separated from the others (Fig 6C and S2 Table; PERMONOVA, *p* = 0.002). Within-treatment variation among samples differed at the end of the treatment period (PERMDISP, *p* = 0.002) and at the end of the recovery period (PERMDISP, *p* = 0.002) because of increased variability among mesocosms in the 80- or 160-day treatments. Differences in dispersion were less obvious, however, in the analysis based on aboveground biomass at the final harvest (PERMDISP, *p* = 0.22).

### Animals

**Fig 7:**
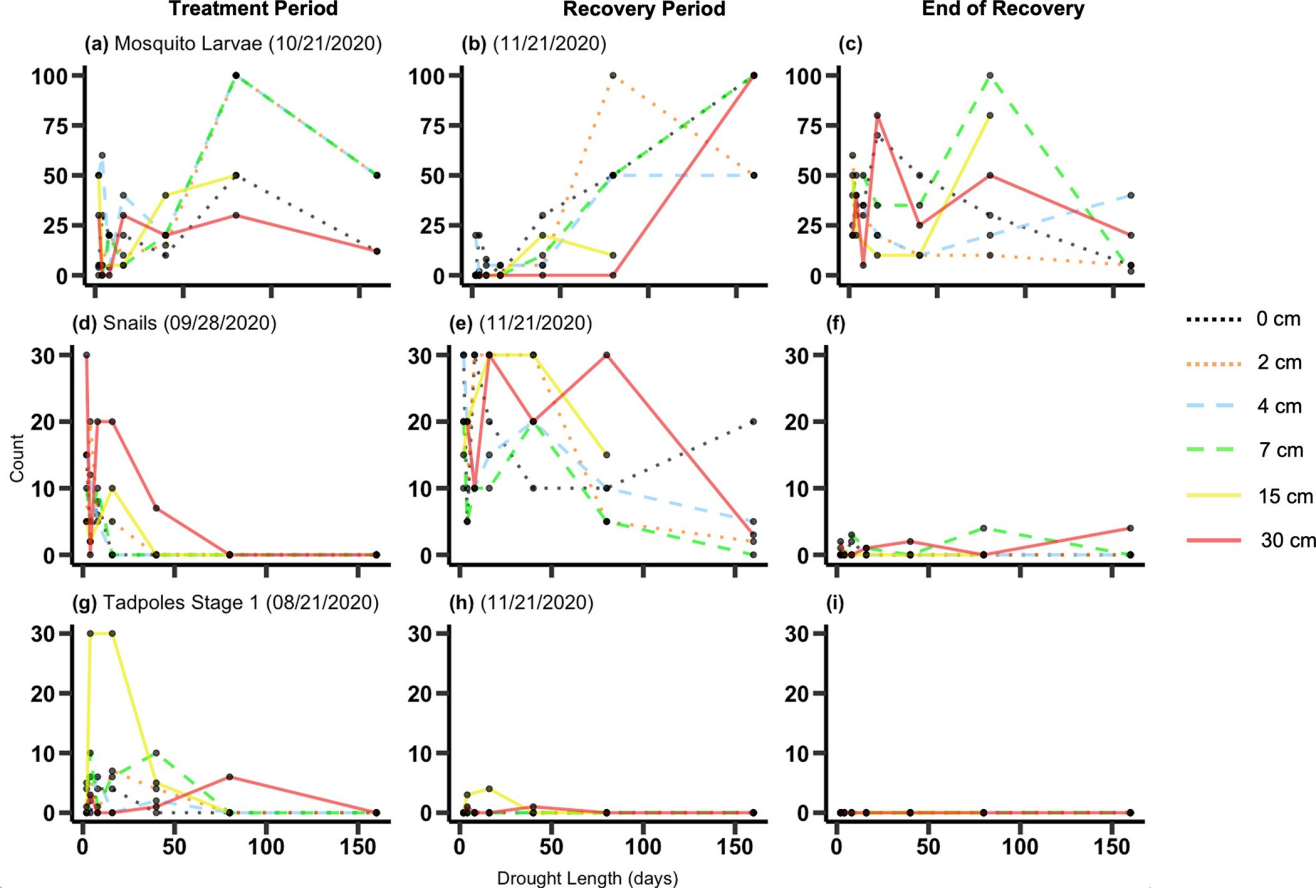
Animal abundance. Animal abundance for mosquito larvae (a-c), snails (d-f) and tadpoles (stage 1) (g-i) as a function of drought length and water depth. Dates chosen for the treatment period and recovery period vary among species to reflect the date when each species was most abundant. The first column of panels shows animal abundance during the treatment period, the second column shows abundance during the recovery period and the third column shows abundance at the end of the recovery period. Statistical results are shown in Table 2.

Mosquito larvae abundance increased with drought length during the treatment period (*p* = 0.01) and during the recovery period (*p*<0.0001) (Fig 7A, 7B and Table 3). The treatments had no effect on the abundance of mosquito larvae at the end of the recovery period after the hard freeze. The abundance of snails increased with higher water depths during the treatment period (*p* = 0.02) and decreased with increasing drought length during the recovery period (*p* = 0.03) (Fig 7D, 7E and Table 3). At the end of the recovery period, after the hard freeze, snail abundance was sharply reduced in all treatments. At this sampling date, drought length interacted with water depth to affect snail abundance (*p* = 0.02), with snails more abundant in treatments that had experienced short droughts with lower water levels or long droughts with deeper water levels (Fig 7F and Table 3). Tadpoles in the first stage (no legs or tail) were only present during the treatment period and early dates in the recovery period, and the treatments had no effect on their abundance (Fig 7G, 7H and Table 3).

**Table 3:** Animal abundance.

**Most Abundant Date During Treatment Period**							
**Species**	**Variables**	**Std. Error**	**t-value**	**R** ^**2**^	***df***	**F**	***p*-value**
Mosquito larvae	Drought Length	0.10	2.58	0.19	36	2.90	**0.01**
	Water Depth	0.50	-0.20				0.84
	DL x WD	0.007	-0.88				0.38
Snails	Drought Length	0.02	-1.96	0.36	36	6.72	0.06
	Water Depth	0.12	2.55				**0.02**
	DL x WD	0.002	-1.30				0.20
Tadpoles (stage 1)	Drought Length	0.03	-1.36	0.08	36	1.04	0.18
	Water Depth	0.13	0.27				0.79
	DL x WD	0.002	0.06				0.95
**Most Abundant Date During Recovery Period**							
**Species**	**Variables**	**Std. Error**	**t-value**	**R** ^**2**^	***df***	**F**	***p*-value**
Mosquito larvae	Drought Length	0.07	7.17	0.73	36	32.32	**<0.0001**
	Water Depth	0.34	-1.50				0.14
	DL x WD	0.005	0.43				0.67
Snails	Drought Length	0.04	-2.26	0.21	36	3.20	**0.03**
	Water Depth	0.18	0.69				0.50
	DL x WD	0.003	-0.02				0.98
Tadpoles (stage 1)	Drought Length	0.003	-0.25	0.10	36	1.32	0.81
	Water Depth	0.02	1.68				0.10
	DL x WD	0.0002	-0.80				0.43
**End of Recovery**							
**Species**	**Variables**	**Std. Error**	**t-value**	**R** ^**2**^	***df***	**F**	***p*-value**
Mosquito larvae	Drought Length	0.09	-1.47	0.06	36	0.78	0.15
	Water Depth	0.45	-0.24				0.81
	DL x WD	0.007	0.81				0.42
Snails	Drought Length	0.004	-0.84	0.25	36	3.82	0.41
	Water Depth	0.02	0.22				0.83
	DL x WD	0.0003	2.39				**0.02**

Results from multiple linear regression analyses of drought length and water depth and their interaction on the abundance of mosquito larvae, snails and tadpoles (stage 1) during the most abundant date during the treatment period, most abundant date during the recovery period and end of recovery. No analysis is shown for tadpoles at the end of the recovery period because they were no longer present. Significant *p*-values <0.05 are shown in bold font. DL: drought length; WD: water depth.

## Discussion

Our results indicate that wetland plant communities near the Texas coast are resistant and resilient to droughts of up to 40 days in duration. Longer droughts modified plant community composition and biomass, but drought length was partially offset by limiting the depth to which water was drained at the onset of the drought. These results suggest that active hydrological control of both natural and artificial wetlands in the area could be used to provide both flood control and wetland function, and that the wetlands would usually not be severely affected by management mistakes in which wetlands were drained but approaching storms failed to materialize.

Our first hypothesis was that extended droughts would alter the composition of the wetland community more than short droughts, because drought tolerance thresholds of wetland plants vary greatly among species [15, 16]. This hypothesis was supported. Several species of plants performed poorly in treatments with long droughts (80 or 160 days) leading to large changes in species composition in those treatments. The species that were most sharply reduced in abundance in longer droughts were *Typha*, *E*. *cellulosa*, *Bacopa* and *Hydrocotyle*. Other species, *E*. *montevidensis*, *Schoenoplectus* and *Juncus*, were not affected by differing drought lengths, and *Ceratophyllum* actually benefitted from longer droughts.

The relative tolerances to drought that we observed for the different plant species were consistent with past work. Other studies have found that *Typha*, *E*. *cellulosa* and *Hydrocotyle* have narrow drought tolerances and are highly sensitive to prolonged droughts in water management strategies that leave wetlands dry for a long period of time [17–19]. In contrast, *Schoenoplectus* and *Juncus* are relatively tolerant to drying out [20, 21]. In our experiment, *Ceratophyllum* benefitted from longer droughts, either because it is poorly adapted to flooding or because it benefitted from reduced competition under these conditions. This is consistent with the fact that *Ceratophyllum* often occurs in shallow, eutrophic environments in which nutrients are elevated to levels unfit for most vegetative species [22–24], leading to a lack of competitors. It was not possible to study all the wetland plants that occur in this area, or in other geographic regions, so additional work would be needed to generalize the results to wetlands containing other plant species.

The animal community was also affected by the drought treatments. Mosquito abundance increased strongly with drought length. An extensive body of research has examined mosquito responses to inundation. In general, they are more abundant in environments with short periods of inundation [25] and with a lack of predators [26, 27]. Adult mosquitoes will often oviposit and emerge in wet periods immediately following prolonged periods of drought to avoid interactions with predators [26]. In contrast, snails were suppressed by long droughts. Freshwater snails are likely to die when their habitats dry out, although some individuals may be able to survive temporary periods of drought buried in the mud [28]. Amphibians opportunistically laid eggs in the mesocosms, and tadpoles were present, but rarely developed past stage 1 (no legs). They may have been limited by some aspect of abiotic conditions or inadequate food in the mesocosms, or by predation, although we did not directly observe predators accessing the mesocosms. Aquatic vertebrates such as larval amphibians and fish would be expected to be highly sensitive to drying out [29]. We did not design our experiment to study vertebrates, and in particular did not stock vertebrates in our mesocosms (amphibians colonized opportunistically). Further experiments would be useful to better assess the effect of variable hydroperiods on vertebrates from this geographic area. In particular, if climate change or wetland management were to alter seasonal patterns of wetland hydrology, this might interrupt or constrain natural patterns of amphibian life cycles [30, 31].

Our second hypothesis was that wetlands that entered into a drought period with a higher initial water level would be more resilient to drought than wetlands that began the drought with little standing water. To test this, the treatments varied in water depth by a total of 30 cm at the beginning of the drought period. This was comparable in magnitude to variation in the water depths of two nearby ponds, in which water depth declined by ~15 cm over ~15-day periods between major rain events that rapidly raised water level by ~20 cm (S3 Fig and S3 Table). This hypothesis was generally supported. For the three plant species that were affected by water depth, all three performed better in treatments that started the drought with a higher water depth. Similarly, snails performed better in treatments that started the drought with a higher water depth. These results make intuitive sense, because wetlands with more water essentially experienced a shorter drought. It took about 30–46 days for tanks flooded to 15 and 30 cm to dry out (S5 Fig). This ~month-long period of slow water decline allowed a grace period during a drought before the wetland completely dried out. In the context of managed wetlands, the amount of water retained as wetlands were drained in anticipation of a storm could be increased if rainfall predictions were uncertain, providing more of a margin for error.

During the recovery period of the experiment, the study system was affected by a severe winter storm, Uri, that swept across Texas from February 11–19, 2021. This winter event brought about snow, sleet and freezing rain along with abnormally low temperatures that persisted for several consecutive days [32]. Ambient air temperatures dropped below freezing on eight days during the recovery period, with three brief dips just below freezing in December 2020 and January 2021 (~-1.5 C), followed by five days with harder and more extended freezes between February 14 and 19, 2021 (~-4 C) (S4 Fig). As a result, all plant aboveground biomass died. However, at no point did the soil temperatures drop below freezing. As a result, plant belowground biomass survived and the plants largely recovered following the freeze (S6 Fig). The animals, in contrast, were all strongly suppressed by the freeze, with snails almost completely disappearing from the mesocosms. In contrast, although mosquitoes were eliminated by the freeze, they readily recolonized afterwards (S7 Fig). Our experiment did not include mesocosms that were protected from the freeze, so we cannot formally evaluate its effects. Functionally, however, it was another severe disturbance, like the droughts, and the wetland communities proved to again be fairly resilient.

We only have data on plant biomass at the final harvest, after the freeze. At this point aboveground dead biomass, which mostly consisted of material that grew during the treatment period and died during the February freeze, was sharply reduced by longer droughts. We hypothesize that we might have seen the same pattern in live aboveground biomass if the freeze had not occurred. At the very least, the data indicate that aboveground biomass was suppressed by drought during the treatment period of the experiment. These results again point to the longer droughts altering wetland structure and function.

Natural wetlands in coastal Texas are seasonal, often drying out, so the plant communities in the area would be expected to be naturally tolerant of droughts [33]. Although we found this to be generally true, longer droughts did affect the plant and invertebrate community, with droughts of 80 and 160 days leading to the largest changes in composition and biomass. Wetland species found in habitats like prairie potholes and playa wetlands that periodically dry out are able to shift their life history traits, in terms of seed bank and seedling recruitment, in order to persist despite extreme changes in hydrology [34, 35]. Nevertheless, the adaptations that allow persistence in these habitats have their limits, and extended periods of water depletion can be detrimental [36]. Over time, plants are able to recolonize even severely affected sites through long-distance dispersal [37–39]. Similarly, wetland insects, which readily re-colonize sites by long-distance dispersal of adults, can be resilient to extended droughts because some individuals survive on the landscape in drought refuges, such as pools that remain wet, wet sediment, and wet leaf litter [40, 41]. In the context of managed wetlands, a small proportion of wetlands across the landscape could be maintained at a higher water level than the others in order to provide a refuge for drought-sensitive species in case of extended droughts. Varying the extent to which different wetlands were drained would also have the desirable effect of increasing beta diversity of wetland communities across the landscape by increasing both habitat heterogeneity and the importance of stochastic colonization processes, thereby increasing species richness at the landscape scale [8, 42].

The relative resilience of Texas wetlands to extended droughts indicates that most aspects of wetland function could recover even if wetlands dry out for extended periods. In other words, wetlands would be resilient to management “mistakes” such as draining a wetland in advance of a predicted storm which later failed to materialize. Significant mistakes (long droughts) might alter the composition of the plant community and exclude some invertebrates, but would not suppress all wetland functions. This is due to the fact that changes in hydroperiod and water depths promote succession in which the plant community shifts from flood-tolerant species to flood-intolerant species, and vice versa [43]. It is likely that droughts of any significant duration would exclude long-lived aquatic organisms such as fish, amphibians and dragonflies; however, this result might be more rather than less natural for this geographic area since the most natural wetlands in the area do dry out periodically. As discussed above, the reliability of the system could be increased by only draining wetlands completely if imminent precipitation was highly likely, and by strategically retaining more water in a few wetlands that could serve as a source of colonists for other sites in the case of an extended drought.

This vision of a network of natural and anthropogenic wetlands that are coupled into a flood control system with significant natural value is achievable with current technology. The accelerated development of automation and wireless and satellite communications allows remote control of a network of water detention structures [44–47]. Continued improvements in forecasting of precipitation will reduce the proportion of “mistakes” when water is released unnecessarily, and water release strategies can be calibrated to different probabilities of immanent precipitation. Recent work has identified optimal strategies for releasing water from a network of wetlands so that releases upstream do not cause floods downstream [48, 49]. Although it would be important to design the system to ensure that it did not increase pollution risks [50], or facilitate the spread of non-native species [51], the end result of implementing such a system would be to greatly increase flood control capacity while at the same time greatly increasing functional wetland area—a rare win-win scenario in the struggle to balance human infrastructure with protection of natural systems.

## Supporting information

S1 Fig Experimental setup. The image shows the forty mesocosms during the growth period. The photograph was taken during winter in January 2020, when aboveground biomass was mostly senescent.
(PDF)

S2 Fig Before and after harvest. The image shows the same mesocosm on May 18, 2021, at the end of recovery period before the harvest (a), and after the harvest on May 19–21, 2021 (b). Aboveground vegetation was harvested from a 0.5 m x 0.5 m quadrat in the center of the mesocosm.
(PDF)

S3 Fig Temperature. Fluctuations in temperature for: a) atmosphere, b) average water temperature across the mesocosms, c) average soil temperature across the mesocosms taken manually and d) average soil temperature across the mesocosms taken with loggers. The timeline for the graphs ranges from just before the start of the treatment period on May 11, 2020 to the end of the recovery period on May 18, 2021. All temperatures depicted are in degrees Celsius. Atmospheric temperature and soil temperature (logger) were recorded every 30 minutes over the duration of the experiment. Water and soil temperatures (manually) were taken weekly during the treatment period and monthly during the recovery period.
(PDF)

S4 Fig Water depth. Actual water depths (cm), taken manually, were plotted as a function of drought length and nominal water depth. Each plot has a light-grey colored box that denotes the start and end of the treatment period for that specific drought length.
(PDF)

S5 Fig Water level. Fluctuations in water level were recorded from two HOBO pressure sensors that were placed in ponds nearby the mesocosm experiment to compare the experimental treatments with the natural hydroperiod. Pressures were corrected for atmospheric pressure recorded by a nearby logger, and corrected to water depth. Data were recorded every 30 minutes from December 2019 to October 2021.
(PDF)

S6 Fig Plant abundance. Plant abundance of *Typha*, *Schoenoplectus*, *E*. *cellulosa*, *E*. *montevidensis*, *Juncus*, *Bacopa*, *Hydrocotyle* and *Ceratophyllum* overtime, averaged across water depths for each drought length. *Ceratophyllum* did not appear until later in the recovery period.
(PDF)

S7 Fig Animal abundance. Animal abundance of mosquito larvae, snails and tadpoles (stage 1) overtime, averaged across water depths for each drought length.
(PDF)

S8 Fig Plant harvest biomass. The biomass (g/m^2^) of all plants were averaged across water depths for each drought length after the recovery period when the final harvest was completed. Aboveground plant biomass was clipped from a 0.5 x 0.5 m quadrat placed in the center of each mesocosm (S2 Fig), and separated by species and whether the sample was living or dead. Belowground biomass was measured by collecting a single monolith (~ 17.5 cm deep, 10 cm wide, 10 cm long) from each tank within the area harvested for aboveground biomass. Aboveground live and aboveground dead bars are overlapping, not stacked (i.e. both bars starting at 0 g/m^2^).
(PDF)

S1 Table Plant biomass at the final harvest. Results from a multiple linear regression analysis of drought length and water depth and their interaction on the aboveground living biomass, aboveground dead biomass and belowground biomass (g/m^2^) summed across species obtained following the harvest. Significant *p*-values <0.05 are shown in bold font.
(PDF)

S2 Table NMDS. Results from two resemblance-based permutation methods, PERMANOVA and PERMDISP, for an NMDS ordination comparing plant abundance across time periods within the experiment (end of treatment period, end of recovery period and final harvest) in Fig 5. The PERMANOVA detects the differences between groups (experimental time periods) while the PERMDISP detects whether some groups are more variable than others. Significant p-values <0.05 are shown in bold font.
(PDF)

S3 Table Abiotic factors. Results from a multiple linear regression analysis of drought length and water depth and their interaction on the various measured abiotic factors. Significant *p*-values <0.05 are shown in bold font.
(PDF)
