# Supplementary material for: Freshwater wetlands for flood control: How manipulating the hydroperiod affects plant and invertebrate communities
Source: PLoS One. 2024 Jul 3;19(7):e0306578. doi: 10.1371/journal.pone.0306578 (PMC11221699; doi:10.1371/journal.pone.0306578)

S1 Fig. Experimental Setup. The image shows the forty mesocosms during the growth period. The photograph was taken during winter in January 2020, when aboveground biomass was mostly senescent.

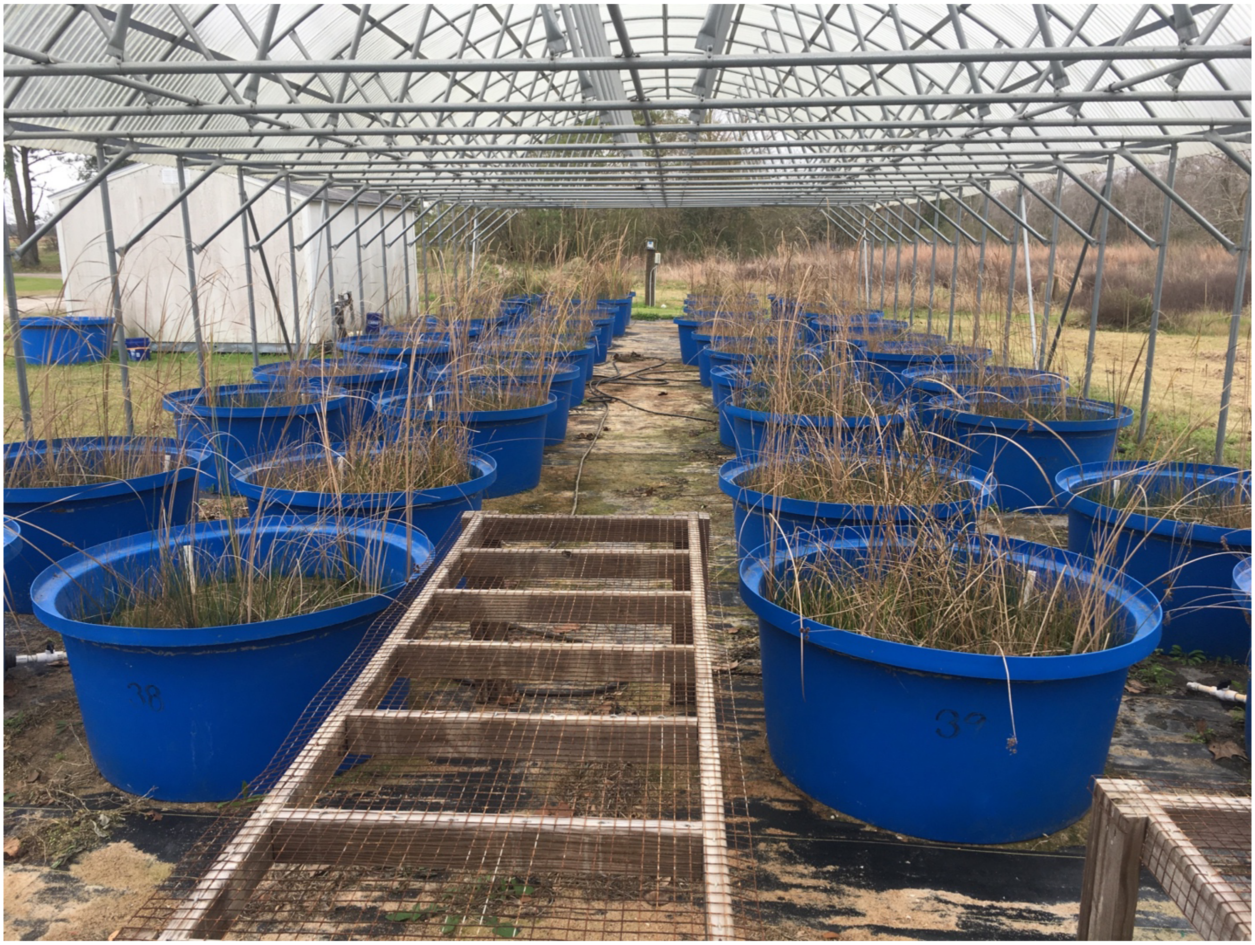

Supplement: S1 Fig — The image shows the forty mesocosms during the growth period. The photograph was taken during winter in January 2020, when aboveground biomass was mostly senescent. (PDF) [file pone.0306578.s001.pdf]
