# Supplementary material for: Freshwater wetlands for flood control: How manipulating the hydroperiod affects plant and invertebrate communities
Source: PLoS One. 2024 Jul 3;19(7):e0306578. doi: 10.1371/journal.pone.0306578 (PMC11221699; doi:10.1371/journal.pone.0306578)

**S2 Fig.** Before and after harvest. The image shows the same mesocosm on May 18, 2021, at the end of recovery period before the harvest (a), and after the harvest on May 19-21, 2021 (b). Aboveground vegetation was harvested from a 0.5 m x 0.5 m quadrat in the center of the mesocosm.

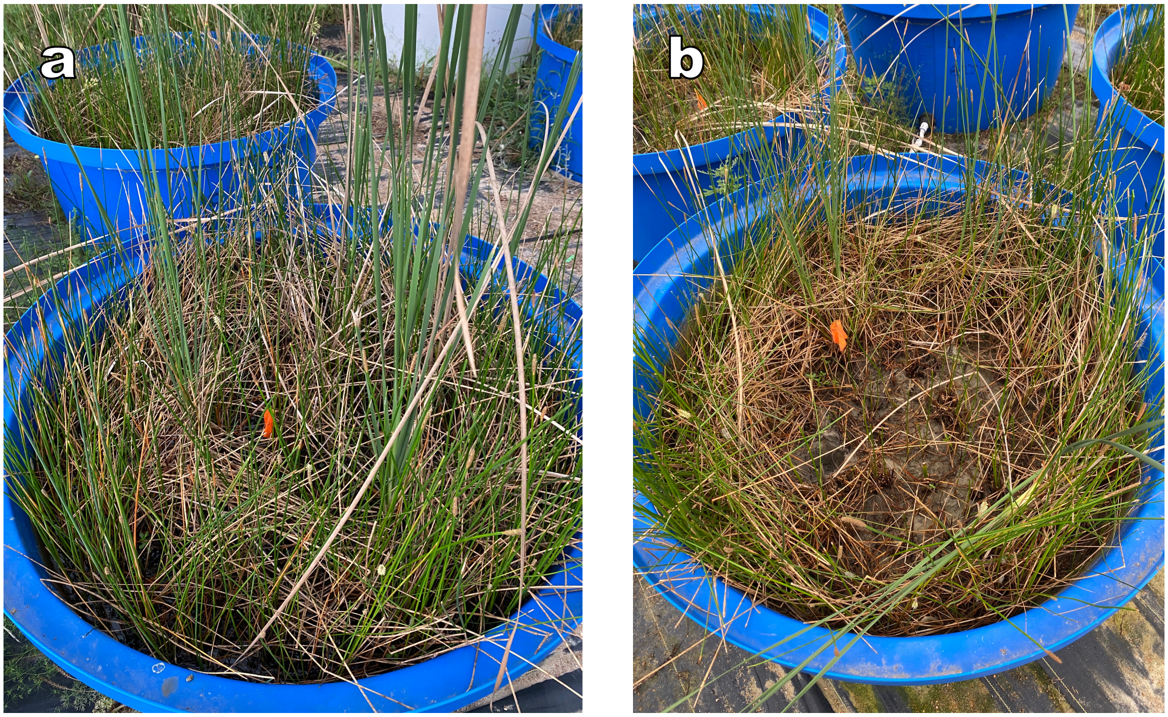

Supplement: S2 Fig — The image shows the same mesocosm on May 18, 2021, at the end of recovery period before the harvest (a), and after the harvest on May 19–21, 2021 (b). Aboveground vegetation was harvested from a 0.5 m x 0.5 m quadrat in the center of the mesocosm. (PDF) [file pone.0306578.s002.pdf]
