# Supplementary material for: Freshwater wetlands for flood control: How manipulating the hydroperiod affects plant and invertebrate communities
Source: PLoS One. 2024 Jul 3;19(7):e0306578. doi: 10.1371/journal.pone.0306578 (PMC11221699; doi:10.1371/journal.pone.0306578)
Supplement: S3 Fig — Fluctuations in temperature for: a) atmosphere, b) average water temperature across the mesocosms, c) average soil temperature across the mesocosms taken manually and d) average soil temperature across the mesocosms taken with loggers. The timeline for the graphs ranges from just before the start of the treatment period on May 11, 2020 to the end of the recovery period on May 18, 2021. All temperatures depicted are in degrees Celsius. Atmospheric temperature and soil temperature (logger) were recorded every 30 minutes over the duration of the experiment. Water and soil temperatures (manually) were taken weekly during the treatment period and monthly during the recovery period. (PDF) [file pone.0306578.s003.pdf]

S3 Fig. Temperature. Fluctuations in temperature for: a) atmosphere, b) average water temperature across the mesocosms, c) average soil temperature across the mesocosms taken manually and d) average soil temperature across the mesocosms taken with loggers. The timeline for the graphs ranges from just before the start of the treatment period on May 11, 2020 to the end of the recovery period on May 18, 2021. All temperatures depicted are in degrees Celsius. Atmospheric temperature and soil temperature (logger) were recorded every 30 minutes over the duration of the experiment. Water and soil temperatures (manually) were taken weekly during the treatment period and monthly during the recovery period.

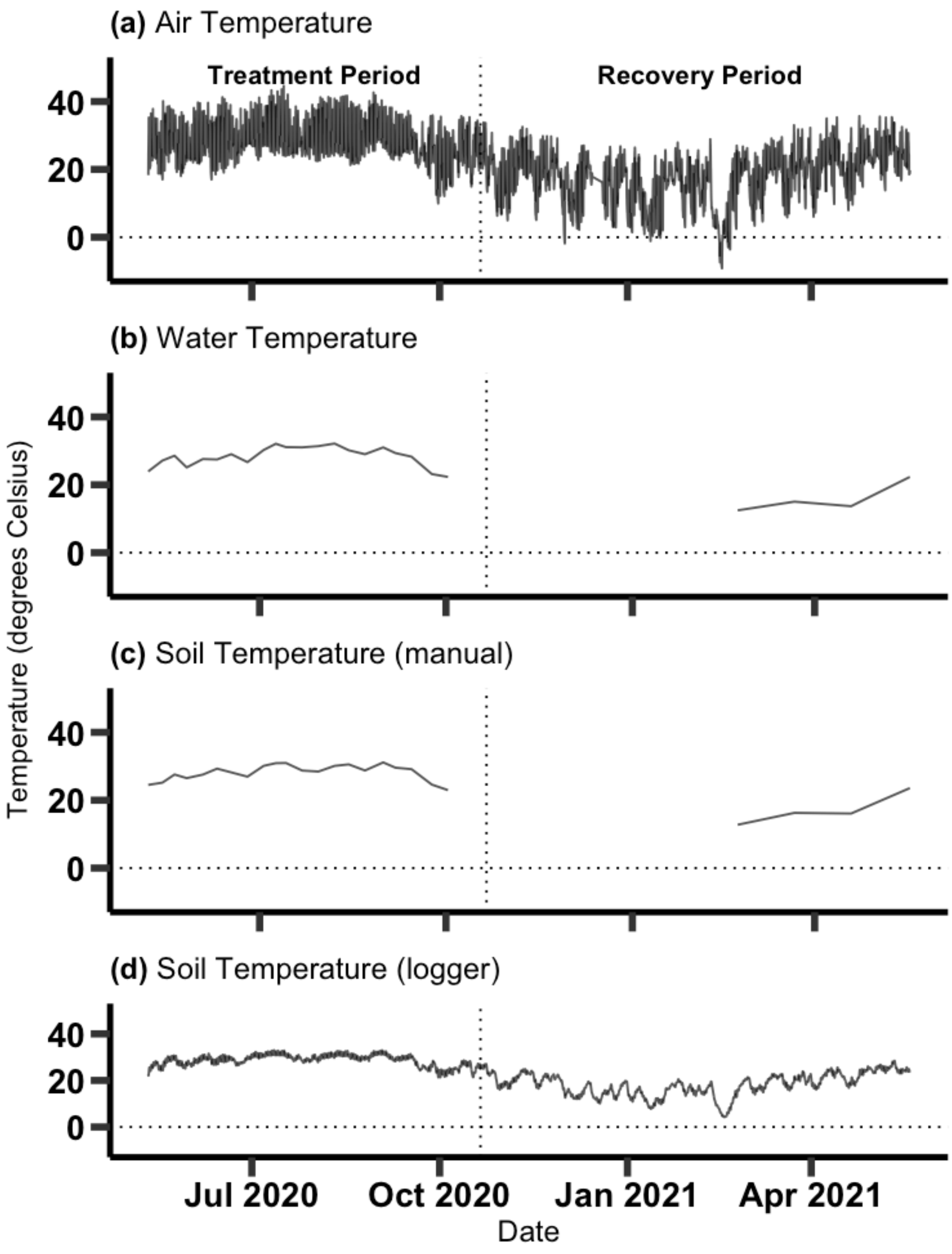

(a) Air Temperature
Treatment Period
Recovery Period
(b) Water Temperature
(c) Soil Temperature (manual)
(d) Soil Temperature (logger)
40
20
0
Temperature (degrees Celsius)
Jul 2020
Oct 2020
Jan 2021
Apr 2021
Date
