# Supplementary material for: Freshwater wetlands for flood control: How manipulating the hydroperiod affects plant and invertebrate communities
Source: PLoS One. 2024 Jul 3;19(7):e0306578. doi: 10.1371/journal.pone.0306578 (PMC11221699; doi:10.1371/journal.pone.0306578)

S4 Fig. Water Depth. Actual water depths (cm), taken manually, were plotted as a function of drought length and nominal water depth. Each plot has a light-grey colored box that denotes the start and end of the treatment period for that specific drought length.

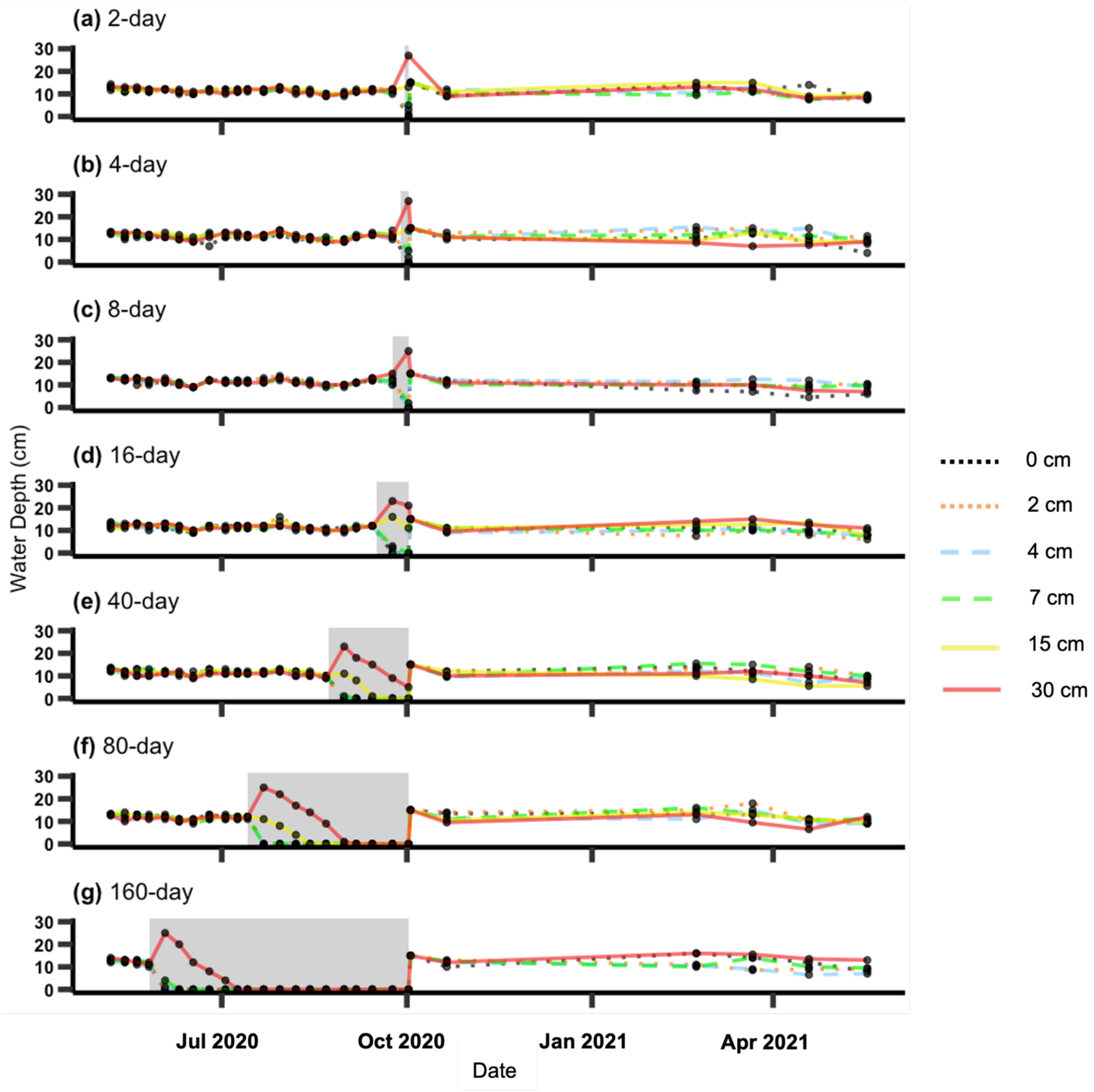

Supplement: S4 Fig — Actual water depths (cm), taken manually, were plotted as a function of drought length and nominal water depth. Each plot has a light-grey colored box that denotes the start and end of the treatment period for that specific drought length. (PDF) [file pone.0306578.s004.pdf]
