# Supplementary material for: Freshwater wetlands for flood control: How manipulating the hydroperiod affects plant and invertebrate communities
Source: PLoS One. 2024 Jul 3;19(7):e0306578. doi: 10.1371/journal.pone.0306578 (PMC11221699; doi:10.1371/journal.pone.0306578)

S5 Fig. Water Level. Fluctuations in water level were recorded from two HOBO pressure sensors that were placed in ponds nearby the mesocosm experiment to compare the experimental treatments with the natural hydroperiod. Pressures were corrected for atmospheric pressure recorded by a nearby logger, and corrected to water depth. Data were recorded every 30 minutes from December 2019 to October 2021.

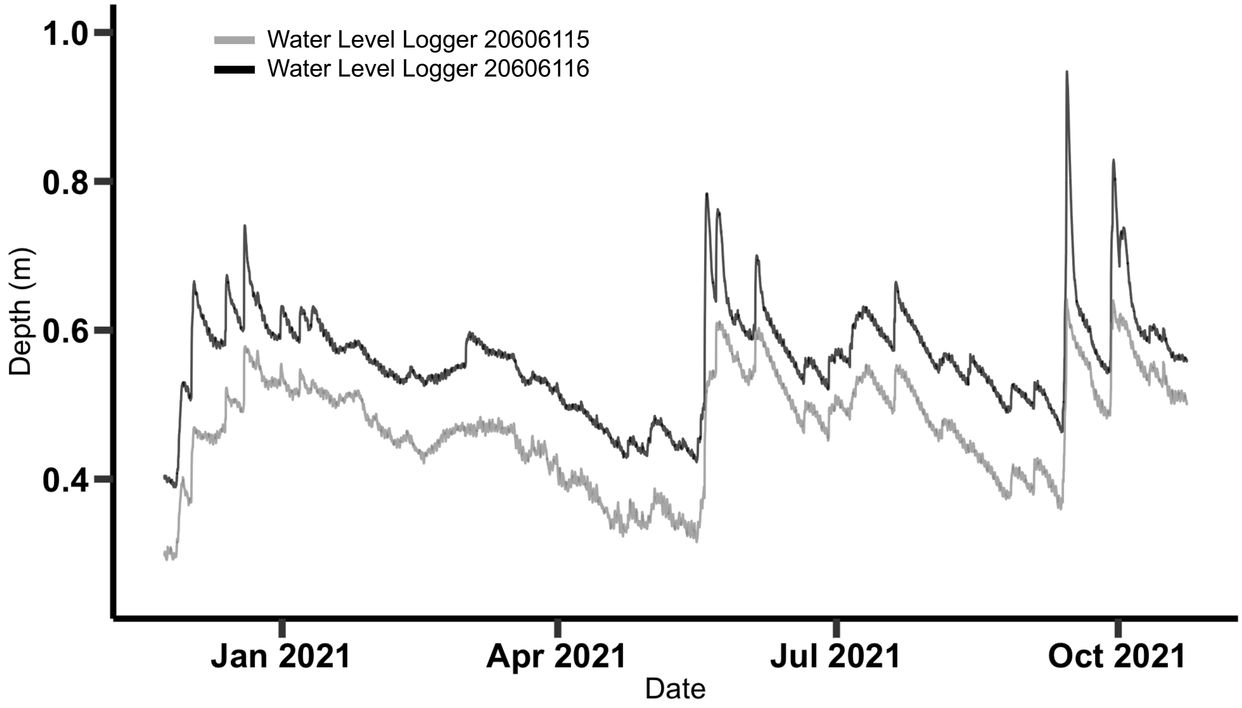

Supplement: S5 Fig — Fluctuations in water level were recorded from two HOBO pressure sensors that were placed in ponds nearby the mesocosm experiment to compare the experimental treatments with the natural hydroperiod. Pressures were corrected for atmospheric pressure recorded by a nearby logger, and corrected to water depth. Data were recorded every 30 minutes from December 2019 to October 2021. (PDF) [file pone.0306578.s005.pdf]
