# Supplementary material for: Freshwater wetlands for flood control: How manipulating the hydroperiod affects plant and invertebrate communities
Source: PLoS One. 2024 Jul 3;19(7):e0306578. doi: 10.1371/journal.pone.0306578 (PMC11221699; doi:10.1371/journal.pone.0306578)

S6 Fig. Plant abundance. Plant abundance of *Typha*, *Schoenoplectus*, *E. cellulosa*, *E. montevidensis*, *Juncus*, *Bacopa*, *Hydrocotyle* and *Ceratophyllum* overtime, averaged across water depths for each drought length. *Ceratophyllum* did not appear until later in the recovery period.

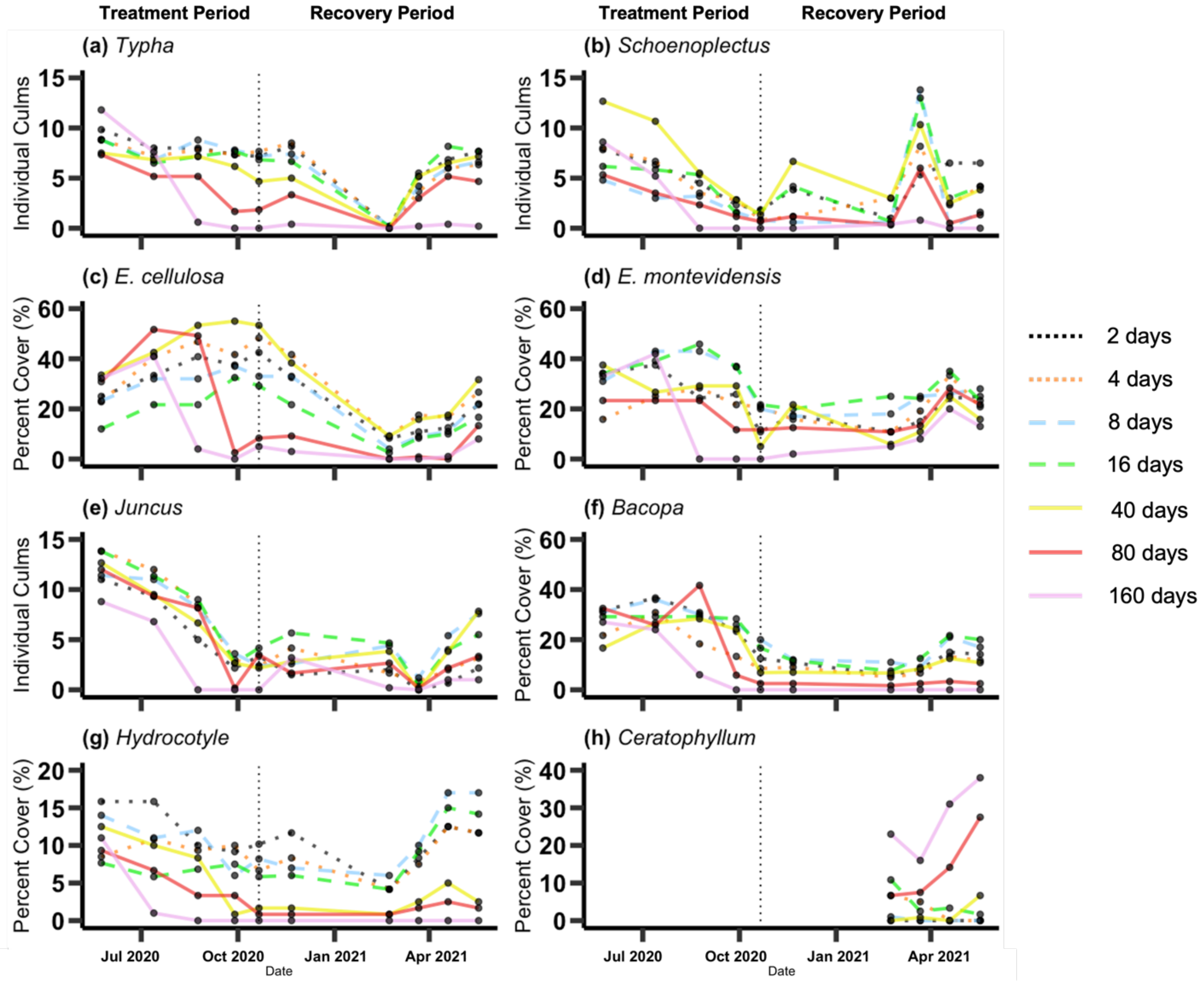

Supplement: S6 Fig — Plant abundance of Typha, Schoenoplectus, E. cellulosa, E. montevidensis, Juncus, Bacopa, Hydrocotyle and Ceratophyllum overtime, averaged across water depths for each drought length. Ceratophyllum did not appear until later in the recovery period. (PDF) [file pone.0306578.s006.pdf]
