# Supplementary material for: Freshwater wetlands for flood control: How manipulating the hydroperiod affects plant and invertebrate communities
Source: PLoS One. 2024 Jul 3;19(7):e0306578. doi: 10.1371/journal.pone.0306578 (PMC11221699; doi:10.1371/journal.pone.0306578)

S7 Fig. Animal abundance. Animal abundance of mosquito larvae, snails and tadpoles (stage 1) overtime, averaged across water depths for each drought length.

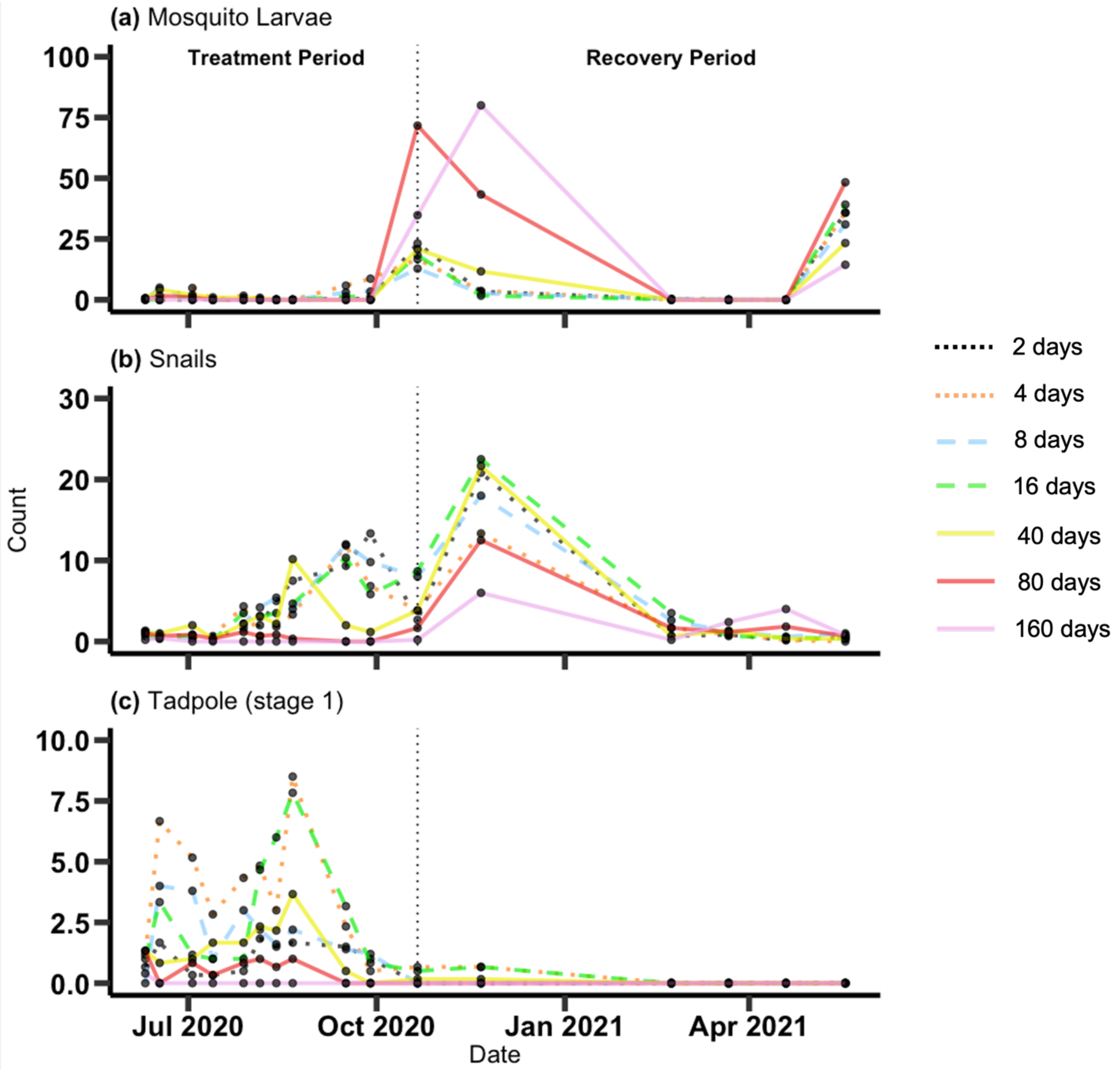

Supplement: S7 Fig — Animal abundance of mosquito larvae, snails and tadpoles (stage 1) overtime, averaged across water depths for each drought length. (PDF) [file pone.0306578.s007.pdf]
