# Supplementary material for: Freshwater wetlands for flood control: How manipulating the hydroperiod affects plant and invertebrate communities
Source: PLoS One. 2024 Jul 3;19(7):e0306578. doi: 10.1371/journal.pone.0306578 (PMC11221699; doi:10.1371/journal.pone.0306578)

S8 Fig. Plant Harvest Biomass. The biomass (g/m^2) of all plants were averaged across water depths for each drought length after the recovery period when the final harvest was completed. Aboveground plant biomass was clipped from a 0.5 x 0.5 m quadrat placed in the center of each mesocosm (Appendix: Figure S2), and separated by species and whether the sample was living or dead. Belowground biomass was measured by collecting a single monolith (~ 17.5 cm deep, 10 cm wide, 10 cm long) from each tank within the area harvested for aboveground biomass. Aboveground live and aboveground dead bars are overlapping, not stacked (i.e. both bars starting at 0 g/m^2).

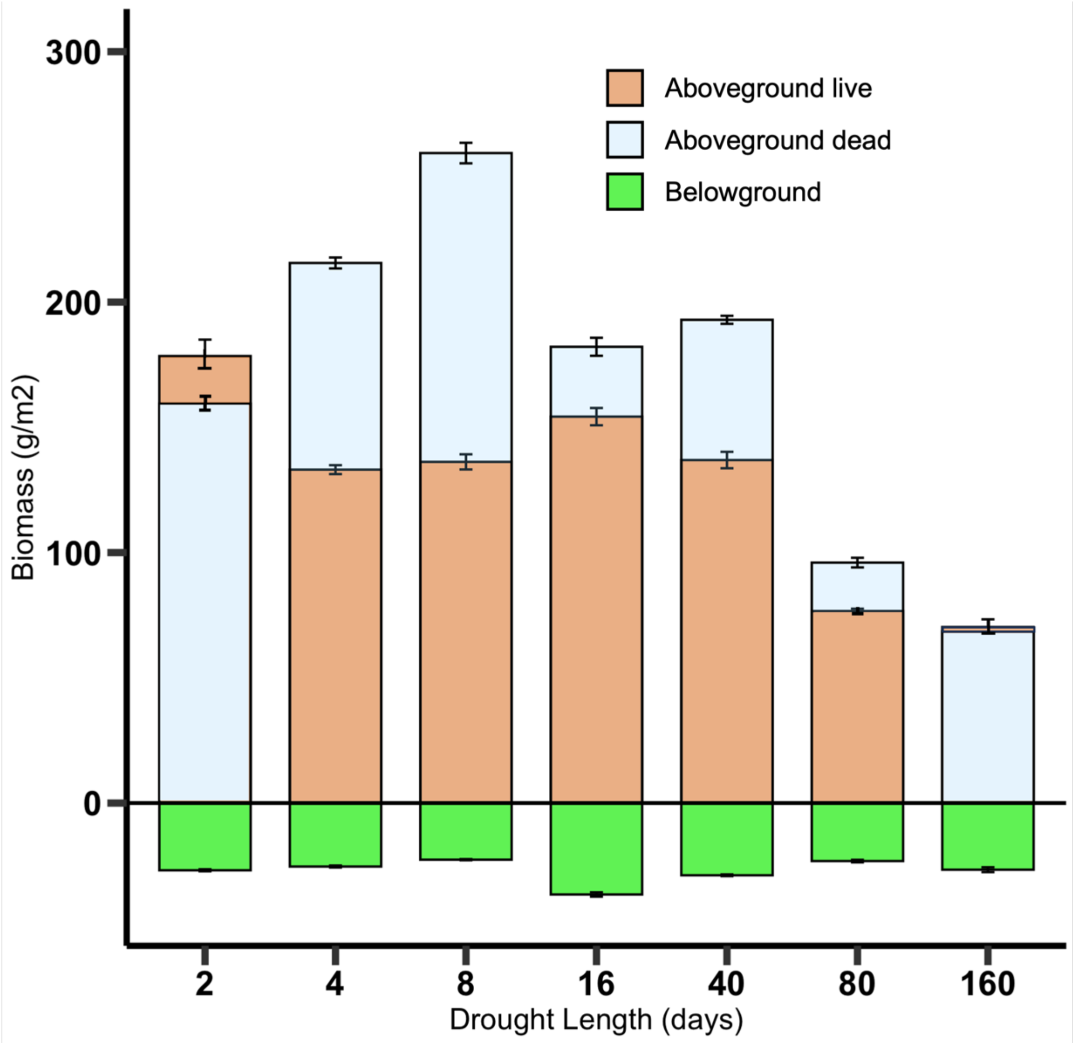

Supplement: S8 Fig — The biomass (g/m2) of all plants were averaged across water depths for each drought length after the recovery period when the final harvest was completed. Aboveground plant biomass was clipped from a 0.5 x 0.5 m quadrat placed in the center of each mesocosm (S2 Fig), and separated by species and whether the sample was living or dead. Belowground biomass was measured by collecting a single monolith (~ 17.5 cm deep, 10 cm wide, 10 cm long) from each tank within the area harvested for aboveground biomass. Aboveground live and aboveground dead bars are overlapping, not stacked (i.e. both bars starting at 0 g/m2). (PDF) [file pone.0306578.s008.pdf]
