# Supplementary material for: Freshwater wetlands for flood control: How manipulating the hydroperiod affects plant and invertebrate communities
Source: PLoS One. 2024 Jul 3;19(7):e0306578. doi: 10.1371/journal.pone.0306578 (PMC11221699; doi:10.1371/journal.pone.0306578)
Supplement: S1 Table — Results from a multiple linear regression analysis of drought length and water depth and their interaction on the aboveground living biomass, aboveground dead biomass and belowground biomass (g/m2) summed across species obtained following the harvest. Significant p-values <0.05 are shown in bold font. (PDF) [file pone.0306578.s009.pdf]

S1 Table. Plant biomass at the final harvest. Results from a multiple linear regression analysis of drought length and water depth and their interaction on the aboveground living biomass, aboveground dead biomass and belowground biomass (g/m^2) summed across species obtained following the harvest. Significant *p*-values <0.05 are shown in bold font.

Biomass at Final Harvest

**Factor**	**Variables**	**Std. Error**	**t-value**	R^2	*df*	**F**	***p*-value**
Aboveground Live	Drought Length	0.38	-1.59	0.11	36	1.47	0.12
	Water Depth	1.89	-0.33				0.74
	DL x WD	0.03	-0.01				0.99
Aboveground Dead	Drought Length	0.31	-2.87	0.31	36	5.47	**0.007**
	Water Depth	1.53	0.65				0.52
	DL x WD	0.02	-0.30				0.76
Belowground	Drought Length	0.05	-1.37	0.27	36	4.37	0.18
	Water Depth	0.27	1.24				0.22
	DL x WD	0.004	1.83				0.08
