# Supplementary material for: Freshwater wetlands for flood control: How manipulating the hydroperiod affects plant and invertebrate communities
Source: PLoS One. 2024 Jul 3;19(7):e0306578. doi: 10.1371/journal.pone.0306578 (PMC11221699; doi:10.1371/journal.pone.0306578)
Supplement: S2 Table — Results from two resemblance-based permutation methods, PERMANOVA and PERMDISP, for an NMDS ordination comparing plant abundance across time periods within the experiment (end of treatment period, end of recovery period and final harvest) in Fig 5. The PERMANOVA detects the differences between groups (experimental time periods) while the PERMDISP detects whether some groups are more variable than others. Significant p-values <0.05 are shown in bold font. (PDF) [file pone.0306578.s010.pdf]

S2 Table. NMDS. Results from two resemblance-based permutation methods, PERMANOVA and PERMDISP, for an NMDS ordination comparing plant abundance across time periods within the experiment (end of treatment period, end of recovery period and final harvest) in Figure 5. The PERMANOVA detects the differences between groups (experimental time periods) while the PERMDISP detects whether some groups are more variable than others. Significant p-values <0.05 are shown in bold font.

**Treatment**		**F**	**R^2**	***p*-value**
PERMANOVA	End of Treatment	4.13	0.44	**0.001**
	End of Recovery	5.55	0.50	**0.001**
	Final Harvest	2.23	0.29	**0.002**
PERMDISP	End of Treatment	2.72		**0.02**
	End of Recovery	3.18		**0.02**
	Final Harvest	1.43		0.22
