# Supplementary material for: Freshwater wetlands for flood control: How manipulating the hydroperiod affects plant and invertebrate communities
Source: PLoS One. 2024 Jul 3;19(7):e0306578. doi: 10.1371/journal.pone.0306578 (PMC11221699; doi:10.1371/journal.pone.0306578)
Supplement: S3 Table — Results from a multiple linear regression analysis of drought length and water depth and their interaction on the various measured abiotic factors. Significant p-values <0.05 are shown in bold font. (PDF) [file pone.0306578.s011.pdf]

S3 Table. Abiotic Factors. Results from a multiple linear regression analysis of drought length and water depth and their interaction on the various measured abiotic factors. Significant *p*-values <0.05 are shown in bold font.

End of Treatment

Factor	Variables	Std. Error	t-value	R^2	*df*	F	*p*-value
Water Depth (cm)	Drought Length	0.005	1.70	0.18	36	2.64	0.10
	Water Depth	0.02	-1.31				0.20
	DL x WD	0.0003	0.02				0.98
Water Temp. (°C)	Drought Length	0.10	1.10	0.32	10	1.58	0.30
	Water Depth	0.02	0.67				0.52
	DL x WD	0.003	-1.43				0.18
Soil Temp. (°C)	Drought Length	0.003	3.26	0.29	36	4.86	**0.002**
	Water Depth	0.02	2.44				**0.02**
	DL x WD	0.0002	-1.52				0.14
Water Content (%)	Drought Length	0.02	-3.14	0.23	36	3.59	**0.003**
	Water Depth	0.12	-0.83				0.41
	DL x WD	0.002	1.26				0.21

End of Recovery

Factor	Variables	Std. Error	t-value	R^2	*df*	F	*p*-value
Water Depth (cm)	Drought Length	0.007	0.05	0.19	36	2.87	0.96
	Water Depth	0.03	-0.01				0.99
	DL x WD	0.0005	1.90				0.06
Water Temp. (°C)	Drought Length	0.001	-3.89	0.39	36	7.64	**0.0004**
	Water Depth	0.006	0.72				0.47
	DL x WD	<0.0001	0.75				0.46
Soil Temp. (°C)	Drought Length	0.002	-3.46	0.39	36	7.56	**0.001**
	Water Depth	0.008	1.28				0.21
	DL x WD	<0.0001	0.12				0.90
Water Content (%)	Drought Length	0.04	-1.50	0.06	36	0.83	0.14
	Water Depth	0.18	-0.87				0.39
	DL x WD	0.003	0.81				0.42
